# A Systematic Review of Toxicity, Biodistribution, and Biosafety in Upconversion Nanomaterials: Critical Insights into Toxicity Mitigation Strategies and Future Directions for Safe Applications

**DOI:** 10.34133/bmef.0120

**Published:** 2025-05-23

**Authors:** Imran Ahamed Khan, Ting Yu, Ming Yang, Jinliang Liu, Zhong Chen

**Affiliations:** ^1^ School of Environmental and Chemical Engineering, Shanghai University, Shanghai 200444, China.; ^2^ Department of Cardiology, Shanghai Sixth People’s Hospital Fujian, Jinjiang, Fujian 362200, China.; ^3^ Department of Cardiology, Shanghai Sixth People’s Hospital Affiliated to Shanghai Jiao Tong University School of Medicine, Shanghai 200233, China.

## Abstract

Upconversion nanoparticles (UCNPs) are emerging as highly promising nanomaterials due to their exceptional optical properties, enabling diverse applications in biosensing, bioimaging, photodynamic therapy, and drug delivery. However, their potential toxicity should be comprehensively investigated for the safe utilization of UCNPs in several biomedical and environmental applications. This review systematically evaluates the current knowledge on UCNP toxicity from 2008 to 2024, focusing on key toxicological pathways, such as oxidative stress, reactive oxygen species (ROS) production, inflammatory responses, and apoptosis/necrosis, alongside their absorption, distribution, metabolism, and excretion processes and kinetics. Distinctively, this review introduces a bibliometric analysis of UCNP toxicity and biodistribution research, providing a quantitative assessment of publication trends, influential authors, leading institutions, funding agencies, and keyword occurrences. This approach offers a macroscopic perspective on the evolution and current landscape of UCNP safety research, a dimension largely unexplored in existing literature. Furthermore, the review combines mechanistic insights into UCNP toxicity with a critical evaluation of surface modifications, physicochemical properties, and administration routes, presenting a holistic framework for understanding UCNP biosafety. By combining bibliometric data with mechanistic insights, this review provides a data-driven perspective on UCNP-associated risks, actionable strategies for enhancing biosafety through surface engineering, and a forward-looking discussion on regulatory challenges and future directions for UCNP-based technologies. These findings bridge existing gaps in the literature and offer a comprehensive resource for researchers, clinicians, and policymakers, facilitating the safe development and utilization of UCNP-based technologies while establishing robust safety guidelines to mitigate adverse effects on human health and the environment.

## Introduction

Upconversion nanoparticles (UCNPs) are mainly utilized in various industries, particularly in biomedicine, because of their exceptional optical characteristics. UCNPs display a phenomenon known as upconversion, where they absorb longer-wavelength photons and emit shorter-wavelength photons, in comparison to ordinary fluorescent materials, which absorb light at shorter wavelengths and emit light at longer wavelengths [1]. The upconversion process in UCNPs comprises the excitation of dopant ions, mainly rare-earth elements such as ytterbium (Yb), thulium (Tm), and erbium (Er), by the absorption of near-infrared (NIR) or infrared (IR) photons [2–4]. The ability of UCNPs to transform low-energy NIR into higher-energy visible or ultraviolet (UV) light has enormous potential for multiple applications. It offers deep tissue penetration and overcomes the drawbacks of tissue autofluorescence. However, UCNPs require surface modification to be compatible with biomedical applications [5]. Modification usually involves coating the UCNPs with a hydrophilic ligand or an additional hydrophilic layer [6]. These characteristics of UCNPs are useful for various biomedical applications, including bioimaging, photodynamic therapy (PDT), synergetic therapies, multimodal biosensing, and drug delivery [7,8]. Despite their potential applications and properties, their safety and toxicity have not been thoroughly understood and addressed. Assessing their biocompatibility, biodistribution, and long-term effects is crucial for the rational design and development of safe nanomaterials, enabling their successful translation from laboratory to clinical applications [9]. In addition, few studies have claimed that surface modification frequently affects UCNP cytotoxicity [10,11]. Therefore, it is necessary to carefully analyze how different surface coatings affect UCNP cytocompatibility [6,12]. This review aims to provide comprehensive knowledge regarding the toxicity of UCNPs and shed light on the factors and mechanisms influencing their toxicity, as well as strategies for minimizing their adverse effects. By addressing the toxicity concerns associated with UCNPs, this review contributes to their safe utilization in biomedical and environmental applications, enabling their transformative impact on healthcare and other related fields.

## Methodology

### Data collection on UCNPs and its toxicity

Web of Science is notable for its thorough and in-depth coverage of scientific papers. It is the most widely used database in bibliometric investigations because it provides general statistics that are necessary for bibliometric software. The Web of Science Core Collection database was used to gather the research findings. The database was selected due to its extensive and comprehensive coverage on UCNPs and its toxicity.

**Fig. 1.**
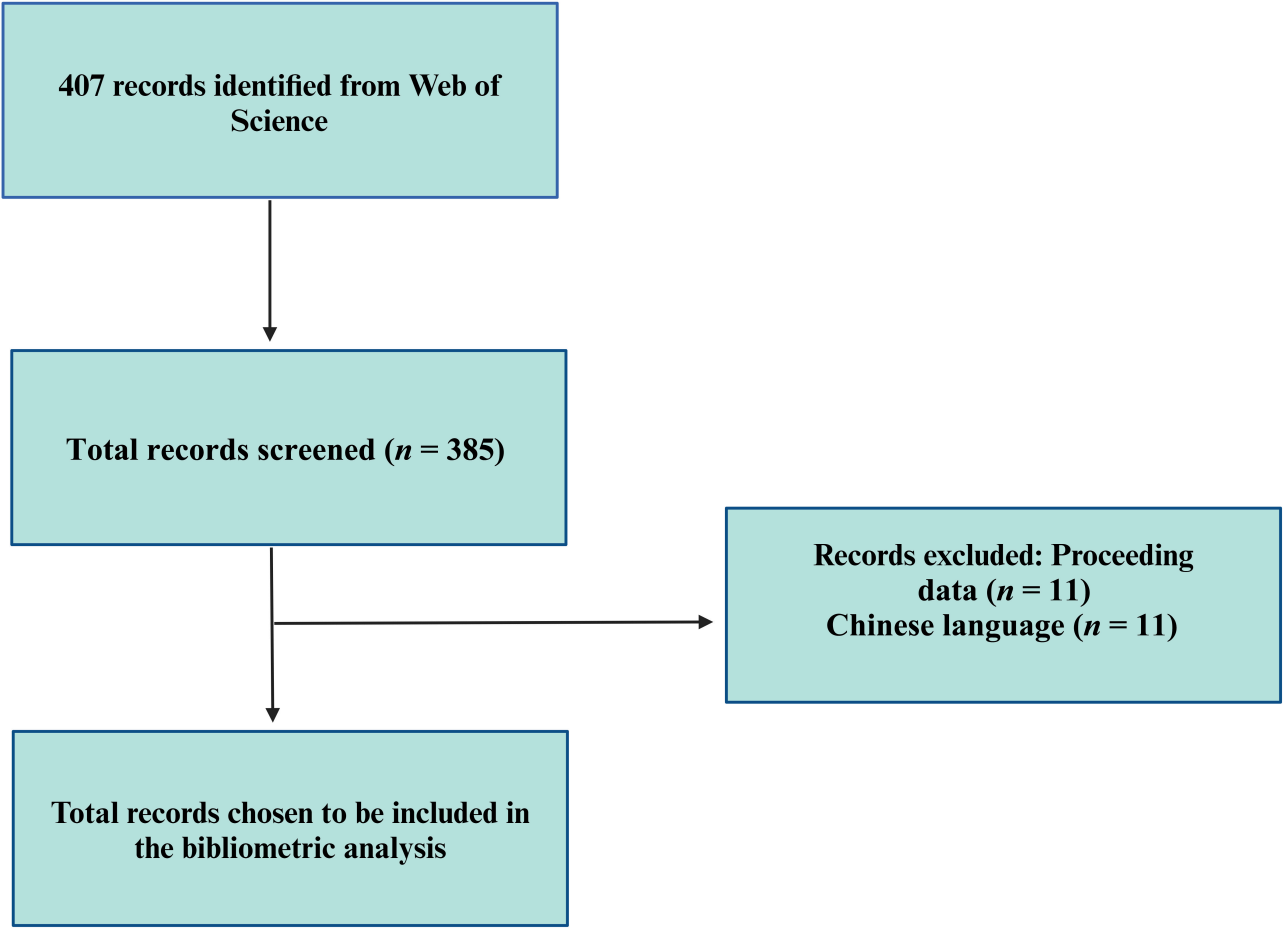
Flowchart for selecting and screening literature.

Using a Boolean operator “TS”, where TS = ((Upconversion nanoparticles) AND (Toxicity)), a systematic search was conducted to gather potentially pertinent research articles. The time frame for the searches was 2008 to 2024. The following were the requirements for inclusion: (a) only research, review, and early access publications were included in the publication category; (b) all entries dealt with the toxicity of UCNPs; and (c) the article language was English. Information about the studies was retrieved, including publication trends, article distribution by countries/institutions, funding agencies, authors, keywords, and prominent journals. Figure 1 shows the procedure for gathering and retrieving the data. The extracted papers were exported as “full record and cited references” and saved in “plain text” format. The data were visualized using VOSviewer.

### Data collection on UCNPs and its biodistribution

Web of Science is notable for its thorough and in-depth coverage of scientific papers, especially on UCNPs and their biodistribution. It is the most widely used database in bibliometric investigations because it provides general statistics that are necessary for bibliometric software.

To find pertinent research publications published between 2008 and 2024, a systematic search was carried out using the Boolean operator “TS” and the query TS = ((Upconversion nanoparticles) AND (biodistribution)). The following were the requirements for inclusion: (a) Only research, review, and early access papers were considered, and (b) the toxicity of UCNPs was covered in all of the chosen entries. The data processing is similar to above (Fig. 1).

## Results

### Search results

The study selection procedure is depicted in Fig. 1. A total of 407 papers were found using the search method. Fourteen records were eliminated following additional screening for the following reasons: Chinese [11] and Proceeding Data [11]. In the end, 385 studies about UCNP and toxicity were chosen to be included in the bibliometric analysis [13–15].

#### The annual paper publication trend

**Fig. 2.**
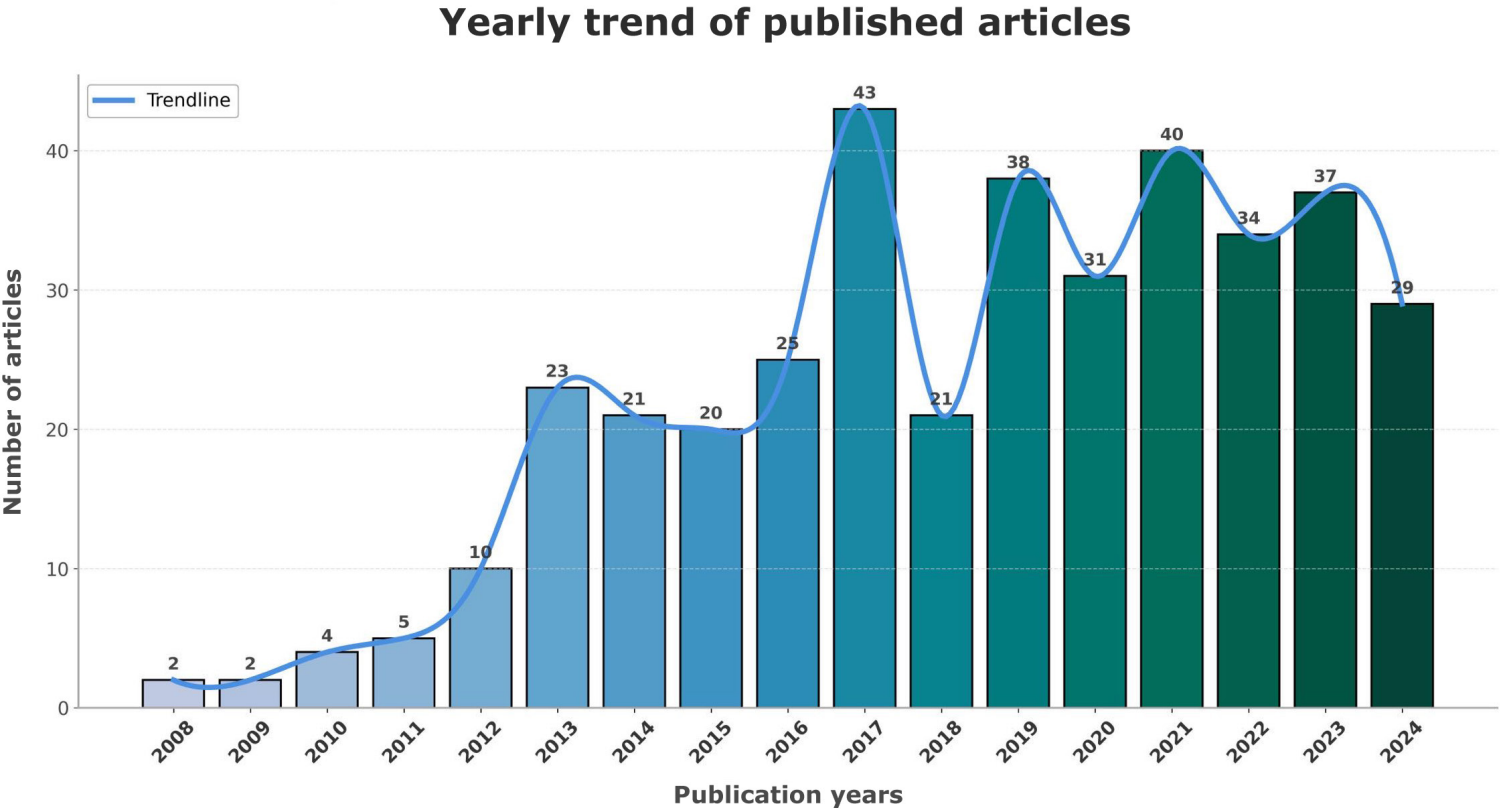
The total number of UCNP and toxicity-related papers published between 2008 and 2024 in the Web of Science database.

A total of 385 papers were published in the Web of Science database between 2008 and 2024. Beginning with just 2 papers in 2008 and 2009, the number of publications was extremely low. Between 2010 and 2017, the number of publications increased steadily, peaking at 43 articles [16,17]. The rise in publications up till 2017 indicates that research on the toxicity of UCNPs is becoming more and more popular. Changes in research funding, focal areas, or field obstacles may be the cause of the post-2017 volatility and recent fall (Fig. 2).

#### Distribution of articles by countries and institutions

**Fig. 3.**
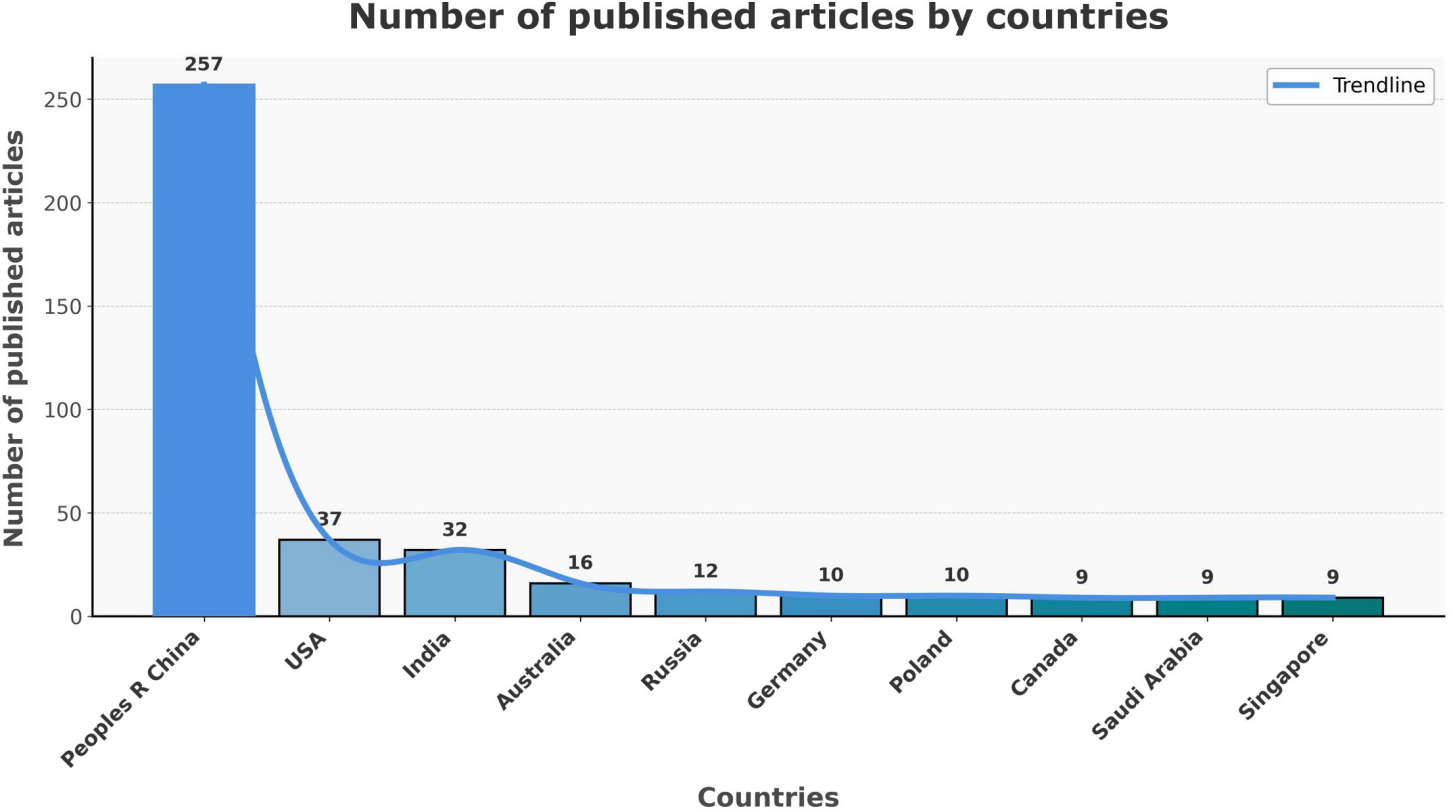
The total number of UCNP and toxicity-related papers published in different countries.

Based on our search parameters, publications about the UCNP and its toxicity were published in 567 institutions in 385 countries. The top 10 countries and universities are displayed in Fig. 3 and Table 1. China has the most published papers, up to 257, accounting for 66.75% of the total; the United States is second, with 37, accounting for 9.61%; and India is third, with 32 articles accounting for 8.31% [18,19]. Among the top 9 universities were the Chinese Academy of Sciences (CAS); Fudan University; Changchun Institute of Applied Chemistry, CAS; University of Chinese Academy of Sciences, CAS; Harbin Institute of Technology University; Jilin University; University of Science Technology of China, CAS; Shanghai University; and Hong Kong Polytechnic University.

**Table 1.** Top 10 institutions and their contribution to the distribution of published articles

Rank	Institutions	Number	% of 385
1	Chinese Academy of Sciences	64	16.623%
2	Fudan University	34	8.831%
3	Changchun Institute of Applied Chemistry CAS	19	4.935%
4	University of Chinese Academy of Sciences CAS	18	4.675%
5	Harbin Institute of Technology University	14	3.636%
6	Jilin University	14	3.636%
7	University of Science Technology of China, CAS	12	3.117%
8	Shanghai University	11	2.857%
9	Hong Kong Polytechnic University	10	2.597%
10	Indian Institute of Technology System (IIT System)	10	2.597%

#### Distribution of published articles by authors

Based on the search results, the 385 studies had 1,952 authors overall, with an average of 5 writers per study. This suggests that the toxicity of UCNPs was a pattern of collaboration among many authors. Out of 1,952 writers, the top 10 with the most publications are displayed in Table 2. With 16 papers pertaining to UCNPs and toxicity, Li FY published the most, followed by Feng W with 13 papers [20].

**Table 2.** Top 10 authors and their contribution to the distribution of published articles

Rank	Authors	Number	% of 385
1	Li FY	16	4.156%
2	Feng W	13	3.377%
3	Lin J	11	2.857%
4	Zhou J	11	2.857%
5	Li CX	10	2.597%
6	Wang Y	10	2.597%
7	Wang J	9	2.338%
8	Zhang Y	9	2.338%
9	Sun Y	8	2.078%
10	Wang L	8	2.078%

#### Distribution of published articles by funding agencies

Based on the selection criteria, top 10 funding agencies out of 47 are represented in Table 3. Among these funding agencies, the Materials Science Multidisciplinary ranked first, accounting for 32.727% of the total. The Nanoscience Nanotechnology ranked second, accounting for 30.649% of the total [21].

**Table 3.** Top 10 funding agencies and their contribution to the distribution of published articles

Rank	Funding agencies	Number	% of 385
1	Materials Science Multidisciplinary	126	32.727%
2	Nanoscience Nanotechnology	119	30.649%
3	Chemistry Multidisciplinary	104	27.013%
4	Materials Science Biomaterials	69	17.922%
5	Physics Applied	66	17.143%
6	Chemistry Physical	56	14.545%
7	Engineering Biomedical	37	9.610%
8	Chemistry Analytical	25	6.494%
9	Physics Condensed Matter	25	6.494%
10	Optics	19	4.935%

#### Occurrence of keyword

**Fig. 4.**
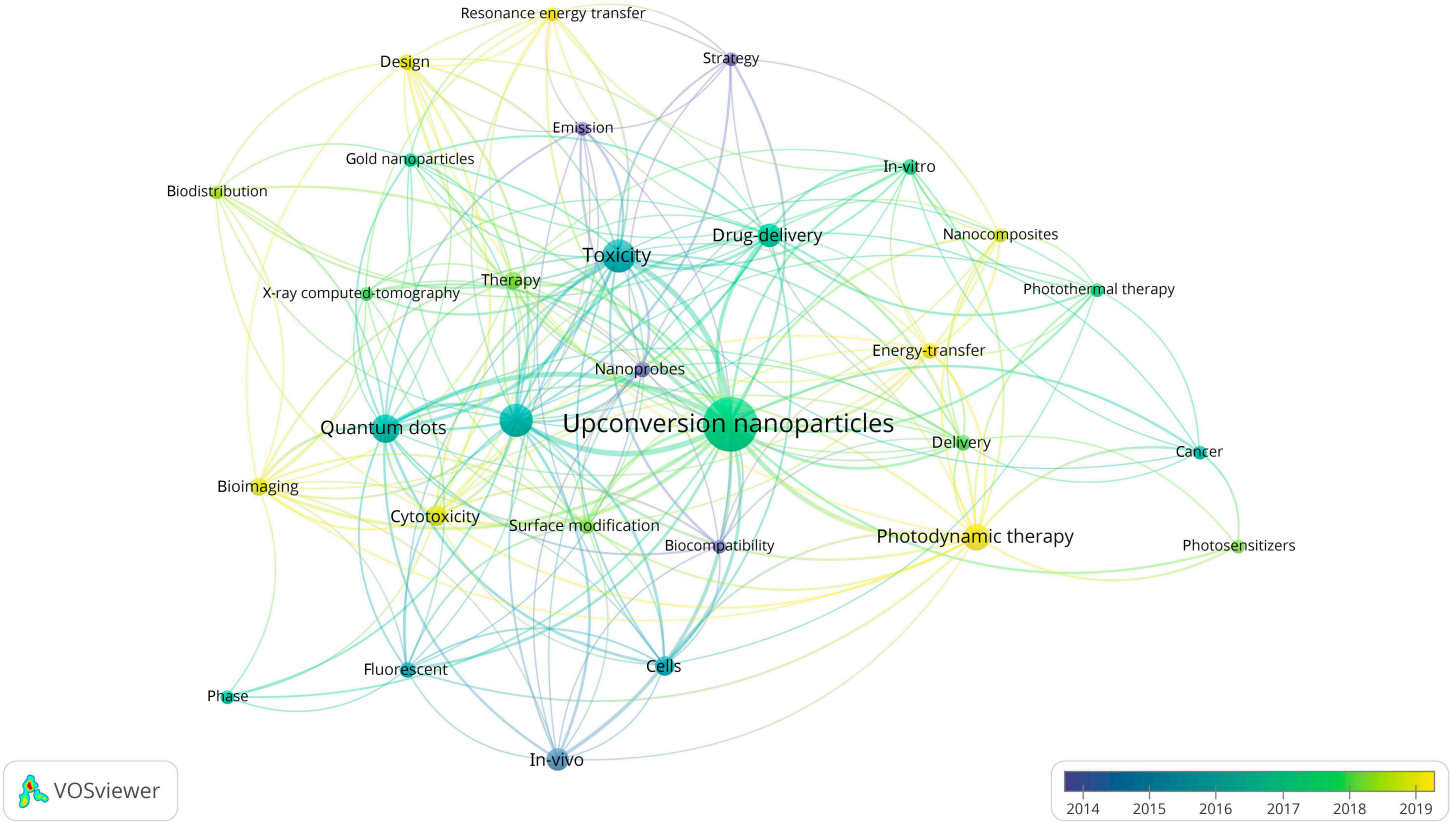
Keyword map of publications on the toxicity of upconversion nanoparticles from 2008 to 2024.

The bibliometric network visualization (Fig. 4) provides a detailed analysis of research trends related to UCNPs and their toxicity interactions. The most common keywords were “upconversion nanoparticles”, “toxicity”, “photodynamic therapy”, “drug-delivery”, “cancer”, and “biodistribution”, reflecting the central themes in UCNP research. The interconnectedness of these terms highlights the multifaceted nature of UCNP studies, particularly in therapeutic and diagnostic applications. The network map reveals strong associations between “photodynamic therapy”, “photosensitizers”, and “cancer”, emphasizing the growing interest in UCNP-based therapeutic strategies. Similarly, terms like “bioimaging”, “nanoprobes”, and “fluorescence” underscore the diagnostic potential of UCNPs, particularly in cancer detection and monitoring. The inclusion of “toxicity”, “biocompatibility”, and “surface modification” highlights the importance of addressing safety and material design considerations to optimize UCNP performance. Emerging trends are evident through terms like “gold nanoparticles”, “quantum dots”, and “nanocomposites”, which suggest interdisciplinary research efforts to develop hybrid systems with enhanced functionalities. The temporal dimension, represented by years 2008 to 2024, shows a progression in research focus, with recent years emphasizing “photothermal therapy” and “in vivo” studies, indicating a shift toward practical biomedical applications. Overall, the network map captures the diversity and interdisciplinary nature of UCNP research, bridging fundamental science and clinical and diagnostic applications. This serves as a valuable tool for identifying emerging trends and guiding future research directions in the field of UCNPs and toxicity [22].

### Search results for data collection on UCNPs and its biodistribution

The process is the same as Fig. 1. The search approach yielded 115 papers. After further screening, 2 records were removed for the following reasons: (a) Correction and (b) Proceeding Data. Ultimately, 112 studies pertaining to the biodistribution of UCNPs were selected for the bibliometric study [23].

### UCNPs and biodistribution: Insights into absorption, distribution, metabolism, and excretion (ADME) processes

**Fig. 5.**
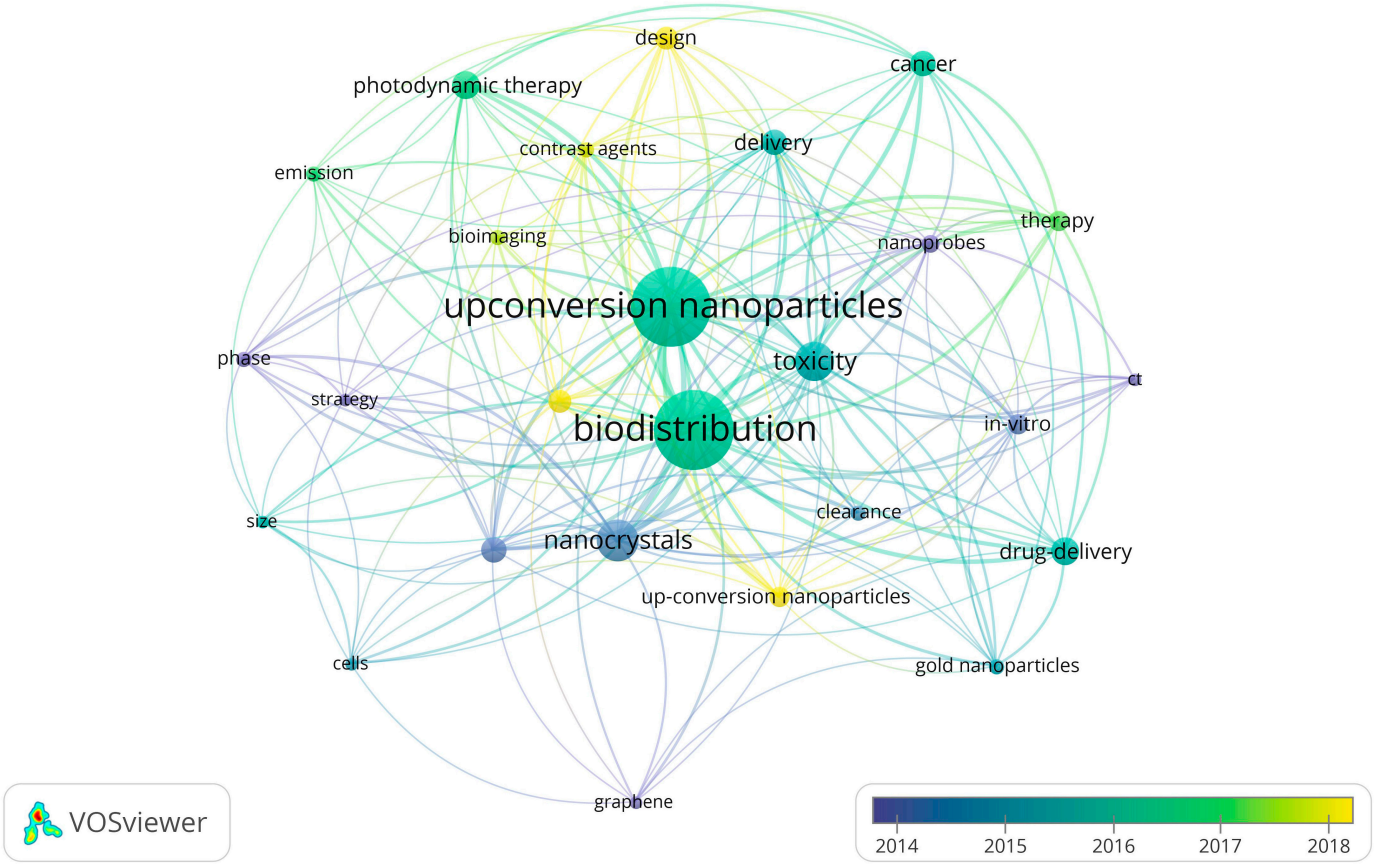
Map of the predominant keywords of studies on upconversion nanoparticles and its biodistribution from 2008 to 2024.

The bibliometric network visualization (Fig. 5) highlights the UCNPs and their biodistribution provides a comprehensive analysis. The graph reveals a rich interplay of terms such as “photodynamic therapy”, “cancer”, “bioimaging”, “toxicity”, and “drug-delivery”, which are closely interconnected, reflecting the multifaceted nature of UCNP research. The network map illustrates the interconnectedness of key terms, with “biodistribution” serving as a central hub linked to critical aspects such as “toxicity assessment”, “pharmacokinetics”, and “protein corona.” These connections emphasize the importance of understanding how UCNPs are absorbed, distributed, metabolized, and excreted in biological systems. Emerging trends, such as “acute hepatotoxicity” and the role of the “protein corona”, further highlight the metabolic and safety considerations associated with UCNPs, providing valuable insights into their biocompatibility and potential organ-specific effects.

The proximity of terms like “bioimaging”, “contrast agents”, and “nanoprobes” underscores the growing interest in leveraging UCNPs for diagnostic applications, particularly in cancer detection and monitoring. Similarly, the strong association between “photodynamic therapy”, “cancer”, and “drug-delivery” reflects the focus on developing UCNP-based therapeutic strategies for targeted treatment and controlled drug release. The integration of these themes with “biodistribution” highlights the critical role of understanding nanoparticle behavior in vivo to optimize their therapeutic and diagnostic efficacy. The network also reveals important material design considerations, with terms such as “size”, “nanocrystals”, and “emission” forming a cluster that emphasizes the tailoring of nanoparticle properties for enhanced performance. Additionally, the presence of terms like “gold nanoparticles” and “graphene” suggests emerging trends in interdisciplinary research, where UCNPs are integrated with other nanomaterials to create hybrid systems with advanced functionalities.

The temporal dimension of the graph, represented by years 2008 to 2024, provides insights into the evolution of research themes over time. For instance, the prominence of “photodynamic therapy” and “bioimaging” in recent years (e.g., 2017 to 2024) indicates a growing focus on theranostic applications of UCNPs. The spatial arrangement of the nodes in the network map reflects the strength of relationships between terms, with closely positioned terms indicating stronger thematic connections. This cooccurrence analysis not only captures the diversity of research themes but also provides a nuanced understanding of the interdisciplinary nature of UCNP studies. By bridging fundamental research with practical biomedical applications, this analysis underscores the pivotal role of UCNPs in advancing fields such as drug delivery, bioimaging, and targeted therapy. The visualization serves as a valuable tool for identifying emerging trends, guiding future research, and fostering collaborative efforts in the development of UCNP-based technologies.

### Related reviews and our contributions

Several review papers have explored the biomedical applications of UCNPs, each with distinct focus. Notable reviews by Oliveira et al., Rostami et al., Del Rosal and Jaque, González-Béjar et al., and Ansari et al. [24–28] highlighted the synthesis, functionalized UCNPs, clinical applications such as bioimaging, phototherapy, and various drug delivery strategies.

Ansari et al. [29] reviewed the historical development of UCNPs to comprehend their synthesis, fundamental studies in biological sciences, and biotoxicity evaluation concerning surface physicochemical characteristics, dimensions, and morphology. Furthermore, they succinctly addressed the methodologies and techniques for assessing the toxicity of nanocrystals (NCs) at the cellular and genetic levels. Their review offered a reference and guidance for UCNPs in biomedical applications. They determined that, although the prospective clinical applications of UCNPs are promising in numerous biomedical fields, it is essential to overcome the related obstacles. Addressing biocompatibility, scalability, targeting efficiency, clinical validation, and ethical considerations is essential for effectively incorporating UCNPs into standard clinical practice. Through these initiatives, UCNPs are poised to contribute substantially to advancements in medicine and health care [29].

Oliveira et al. [24] published a review assessing the current status of nanotoxicity research on UCNPs in comparison to other nanomaterials and outlined pathways for the clinical translation of UCNPs. They concluded that it is imperative to direct the advancement of novel UCNP-based biomedical applications through time- and cost-effective methodologies and to assess the effectiveness, quality, and safety (EQS) of UCNPs in authentic biological contexts. These activities will close the translational gap between research facilities and medical and biological environments, facilitating improved and safer theranostic applications of UCNPs [24].

In their review, Rostami et al. [25] highlighted UCNPs’ physical and optical features and showed their prominent applications in bioimaging, theranostics, cancer therapy, and optogenetics. To better comprehend UCNPs’ potential for theranostic usage, they summarized their physical/chemical features and photoactivity behavior, including upconversion mechanisms, crystal compositions, and efficient synthesis methods. After that, this review focused on using these materials in medicine for delivering, targeting, tumor therapy, and bioimaging in vitro and in vivo. Discussions in this review article showed that the unique properties of modified UCNPs in photodynamic treatment (PDT) by excitation of NIR light is a better tool that could become a surefire and painless way to get rid of cancerous cells. Furthermore, these particles’ comparatively high surface area-to-volume ratio for loading chemicals and biomaterials makes them a suitable platform for medication and gene delivery for various disorders that require precise and targeted targeting. These functionalized fluorophore probes are essential for in vitro/in vivo imaging with high sensitivity and deep stimulator penetration due to the rapid absorption of UCNPs by cells and tissues. They detailed how the UCNPs’ unusual optical features and novelty in biochemistry and medicine make most commercial imaging equipment unsuitable for direct use with them. Usually, confocal microscopes use visible light sources and UV. However, the NIR sources of in vivo imaging machines are not focused or strong enough to excite UCNPs. This is why scientists must use sources like continuous wavelength lasers to make their own imaging tools. Recent reports show how these novel NPs of different substances can replace mainstream methods that are difficult to detect and target, especially in hard-to-reach areas like deep brain structures. They concluded that the potential of adaptable UCNPs can be harnessed successfully when developing contemporary and more individualized therapeutic approaches.

In their review, Gorris and Resch-Genger [30] highlighted and discussed the pros and cons of employing UCNPs as background-free luminescent carriers in bioimaging and bioanalytical tasks. They advocated the preparation of safe nanoparticles before any life-science application. Förster resonance energy transfer (FRET) is used for many bioanalytical detection methods. However, FRET for UCNPs is still up for question and needs to be improved. The necessity for standardized and dedicated instruments and recent research on the potential toxicity and dissolution of UCNPs is discussed. Toward the end of the review, they additionally detailed the upcoming developments and difficulties encountered in UCNPs [30].

In 2013, Gu et al. [31] presented an overview of recent progress in making UCNPs that have the right size, better and more controllable upconversion luminescence, and multiple functions all at the same time. They also address the chemical processes used to modify the surface of UCNPs so that they are safe and soluble in water. Finally, they discuss some examples of how UCNPs are used for in vivo bioimaging, NIR-triggered drug/gene delivery, and PDT. In these perspectives, the authors explain more about how it is necessary to structure nanotoxicology data to make smart designs of UCNP materials and use their surface chemistry in safer medicinal settings. According to their conclusions, UCNPs can offer a perfect multifunctional platform for addressing several important problems in the field of medicine, including multimodality medicine, personalized therapies, and theranostics [31].

However, these publications allocate only one subsection to toxicity considerations. Additionally, a few overview studies have addressed the biocompatibility of UCNPs for cancer and tumor therapies, but toxicity remains a secondary focus [32–34]. To our knowledge, there are merely 2 review articles exclusively dedicated to exploring the toxicity of UCNPs [35,36]. Given the limited coverage of toxicity-related aspects in the existing literature, our proposed review aims to shed light on comprehensive analysis encompassing the in vitro and in vivo toxicity, biodistribution, mechanisms of action, and biosafety of UCNPs. The uniqueness of our review lies in its explicit emphasis on the toxicity of UCNPs and related concerns, addressing current trends, and providing a comprehensive summary of the topic, while maintaining a critical perspective on the existing literature. By addressing these critical aspects, our study strives to bridge the existing gap and substantially contribute to understanding UCNP toxicity and biosafety.

## Physicochemical Properties of UCNPs

### Synthesis approaches of UCNPs

Good quality UCNPs are important to meet the demands of biomedical applications. UCNPs have 3 components: sensitizer, host matrix, and activator. To ensure maximum radiative emission and minimal nonradiative losses, an ideal host matrix should possess low lattice photon energies [37]. Among the different types of fluorescent materials, those containing Ln^3+^ ions offer distinct advantages for fluorescent-based technologies. These materials possess chemical and thermal robustness, unique optical properties, superior photostability, narrow absorption, luminescence transitions, good biocompatibility, remarkable quantum yield, absence of auto-fluorescence, extended decay time, and low toxicity [38].

The synthesis and characterization of UCNPs are essential to tailor their properties and optimize their performance for specific applications. Synthesis methods determine the size, shape, composition, and crystal structure of UCNPs, which directly affect their optical properties and stability [39–41]. Various approaches for the synthesis of UCNPs, such as microwave-assisted heating, thermal decomposition, hydrothermal/solvothermal, and coprecipitation, offer control over these parameters, enabling the customization of UCNPs with desired characteristics [9].

The thermal decomposition approach has been widely applied in the synthesis of high-quality UCNP crystals, including NaYF_4_, LiYF_4_, NaGdF_4_, and NaLuF_4_ [42–46]. However, the thermolysis of trifluoroacetates at elevated temperatures generates toxic fluorinated and oxyfluorinated carbon species [46]. This necessitates a properly ventilated reaction environment and reduces toxic substances released during the entire procedure, thereby raising safety concerns. To address these issues, recent efforts have focused on the rational design of UCNPs with the anticipated morphologies under controlled thermal decomposition. This is achieved by varying parameters, such as additives, ligands, reaction time, and temperature [45].

Another solution-based approach is hydro/solvothermal synthesis, in which NCs are produced at high temperatures and pressures in an aqueous solution within a tightly sealed reaction container [46]. Hydrothermal synthesis offers convenient control over experimental parameters, such as pH, fluoride precursor source, molar ratios, and the addition of ligand agents, such as citric acid, ethylene diamine tetraacetic acid, and cetyltrimethylammonium bromide [47]. Using this approach, several rare-earth doped NCs, such as NaYbF_4_, NaYF_4_, carbon-coated NaLuF_4_, NaGdF_4_, CaF_2_, and LnF_3_ (Ln = La, Ce, and Pr) have been successfully synthesized [48–50]. Notably, Liu et al. reported the hydrothermal synthesis of dual-color-banded β-NaYF_4_ microrods doped with different activators at their tips [9,51]. Chemical coprecipitation is another convenient method for synthesizing UCNPs [52]. This method does not involve harsh reaction conditions and requires only a precursor solution for the precipitation of the desired products. Martin et al. [53] pioneered synthesizing crystalline NaYF_4_:Yb, Pr UCNPs using a coprecipitation technique at a low temperature of 80 °C. Another promising method is the microwave-assisted heating method, in which reactants are rapidly heated upon exposure to electromagnetic waves [52,54]. This approach offers advantages such as reduced reaction time, lower energy consumption, and green synthesis. These different synthesis approaches offer researchers various options to tailor the properties and morphology of UCNPs, enabling advancements in upconversion nanotechnology.

The UCNP synthesis approaches tend to influence the toxicity and bioaccumulation profiles of UCNPs significantly. Minimizing toxicological concerns in biomedical applications, including drug administration, imaging, and therapies, requires an understanding and commitment to improving these synthesis techniques. Since the toxicity of UCNPs has been shown to be concentration-dependent, the dosage should be reduced. Consequently, additional optimization of the quantum yield is necessary. The short excitation cross-sections of lanthanide ions and nonradiative decay are the primary factors influencing upconversion efficiency; hence, increasing the size, altering the crystal field symmetry, and laser annealing can all increase upconversion efficiency [55,56]. Additionally, the environment’s lattice structure, doping levels, and surface conditions all have an impact on UCNPs’ upconversion efficiency [57]. The safety profile of UCNPs for long-term usage in human health applications can be enhanced by customizing the synthesis process to achieve the required size, surface characteristics, stability, and dispersibility [58].

### Surface modifications and functionalization

Functionalization improves the biocompatibility, targeting ability, and imaging contrast of UCNPs by adding specific chemical or biological properties. Despite the many advantages and numerous biomedical applications of UCNPs, there is a growing need for their biosafety and potential adverse effects [5,24]. One significant concern arises from the dissolution of UCNPs, which release fluoride and lanthanide ions [24,59,60]. These ions are potentially cytotoxic in biological systems, mainly under high-dilution conditions in aqueous media [60,61]. Specifically, fluoride ions inhibit mitochondrial activity, cell growth, protein synthesis, and proliferation in cultured human pulp cells [62]. To address this issue, surface modification techniques can be used to improve the properties of UCNPs and make them more suitable for various applications [63,64]. Such modifications improve the colloidal stability and dispersibility and reduce the dissolution of UCNPs in aqueous environments [65,66]. These strategies enhance biocompatibility and reduce potential adverse effects on biological systems by decreasing the discharge of fluoride and lanthanide ions. Surface modification improves the stability of UCNPs in aqueous solution and protects them from aggregation and degradation. Several methods can be used to modify or functionalize the surface of UCNPs.

Ligand exchange is a commonly used method that involves replacing the surface ligands of UCNPs with ligands of different properties. The effectiveness of 5 different surface capping ligands, namely poly (acrylic acid), polyallylamine, citrate, phosphonoglycine, and polyethylene glycol (PEG), was investigated to provide long-term colloidal stability to NaYF_4_ (Yb, Er) UCNP [67] Several studies have also established the dispersion stability and biocompatibility [68–70]. Another study used a binary ligand strategy to demonstrate the photochromic modulation of lanthanide-doped UCNP–spiropyran (SP) conjugates. By employing an SP-to-oleate ligand exchange reaction, it was possible to precisely control the amount of SP ligand on the surface of UCNPs, thus allowing for the regulation of steric congestion within the ligand layer. Introducing a highly congested SP layer prolongs the lifetime of the purple open-ring state and enhances the red fluorescence response under UV excitation [71]. Other covalent modifications involve the chemical bonding of functional groups on the surface of the UCNPs. The choice of method depends on the specific UCNP and the desired properties.

## Factors Influencing the Toxicity of UCNP

To comprehend the potential biological consequences of UCNPs, it is important to consider all variables affecting their toxicity. The following are some of the main elements that affect UCNP toxicity:

### Composition and surface chemistry

The composition of UCNPs, including the selection of host and dopant materials, can affect their toxicity and surface chemistry, including surface coatings or functionalization, and can also affect the interaction of UCNPs with biological systems and, subsequently, their toxicity [35,51]. These interactions may cause internal cellular damage, oxidative stress, ion imbalance, membrane disruption, and cell death. These interactions and the resulting toxicity are mediated by many important factors, including surface charge, size, shape, and surface functionalization. Designing UCNPs with enhanced biocompatibility for use in biomedical domains requires understanding and controlling these parameters. The most frequent interactions, known as electrostatic interactions, between the negatively charged cell plasma membrane and the charged UCNPs have a significant impact on the cytotoxicity and uptake efficiency of UCNPs [72]. The lanthanide elements used in UCNPs, such as gadolinium (Gd) or ytterbium (Yb), have been associated with potential toxicity [58,59]. Saleh et al. [10] assessed the protective effects of various surface coatings on NaYF_4_:Yb^3+^, Er^3+^ UCNPs.

Several studies have provided novel insights into the role of surface chemistry in the stability, dissolution behavior, and cytotoxicity of UCNPs. Bastos et al. [11] investigated NaYF_4_:Yb, Er UCNPs, using 4 different bound surface ligands, i.e., citrate, alendronate, ethylenediamine tetra (methylene phosphonate) (EDTMP), and poly (maleic anhydride-alt-1-octadecene) (PMAO), along with 2 different thicknesses of silica coatings. The release of fluoride ions and cytotoxicity to human keratinocytes was investigated in parallel with evaluating the cytotoxic effects of the ligands, sodium fluoride, and lanthanide ions. Cytotoxicity studies of UCNPs with different surface modifications demonstrated that the biocompatibility of EDTMP-UCNPs and PMAO-UCNPs aligns with the minimal release of fluoride ions from these samples. UCNPs with a sufficiently thick silica shell effectively prevent UCNP dissolution and release of cytotoxic ions, along with low cytotoxicity [65]. Several reviews have been published on the effects of surface modification of frequently used UCNPs on cytotoxicity and other bioapplication problems [6,38].

Guller et al. [12] explored a solution by investigating additional surface alterations to UCNP@PEI to produce less toxic and functional nano-theranostic materials. Five of the six types of multilayer polymer coatings developed to cover the initial UCNP@PEI surface reduced the cytotoxicity to human skin keratinocytes after exposure for 24 and 120 h. The lifespan and photoluminescence spectra of surface-modified UCNP@PEI were also evaluated. These findings demonstrate that each coating in biologically relevant aquatic environments has a different effect, despite reducing cytotoxicity as the exterior polymer coatings of UCNP@PEI quench the upconversion photoluminescence. To simplify the clinical application of such nanoparticles, an optimized technique for the rational surface design of UCNP@PEI under biologically relevant circumstances has also been proposed [12].

The primary phenomena behind the toxicity potential of UCNP are oxidative stress and ROS generation. The toxicity caused by oxidative stress was regularly examined in the majority of the published publications [73,74]. There are numerous ways to generate ROS. Utilizing the surface groups of Ln^3+^ metals or UCNPs in one-electron oxidative reactions is one method. It is important to remember that the external area of an NP is rather large compared to its volume. Increases in surface chemistry typically accompany increases in porous structure, and this can lead to an increase in ROS generation. ROS can also be produced by mitochondrial activity and the subsequent release of ROS into the cytoplasm through holes in the inner membrane of the mitochondria caused by UCNPs. In healthy tissue, the ratio of ROS to cytoplasmic antioxidants is maintained [75]. By increasing the concentration of ROS or decreasing antioxidant capacity, chemicals are present in cells because of the oxidative stress in the form of ROS, which overwhelms the cells’ defenses [76].

### Particle size and shape

The size and shape of UCNPs play crucial roles in determining their toxicity [77]. Smaller nanoparticles tend to have higher surface area-to-volume ratios, which can enhance their reactivity and potential biological interactions [9,78]. Similarly, the shape of UCNPs can affect their cellular uptake, biodistribution, and biological responses [9,54]. Their size, shape, and surface modification primarily determine the toxicity of UCNPs in biological systems. UCNPs may cause increased toxicity by modulating changes in LDH and ROS levels or by triggering inflammatory responses. For biomedical applications like drug delivery, UCNPs must be carefully designed to minimize toxicity while optimizing their function, considering their size and surface characteristics. The findings of a systematic investigation using microwave-assisted synthesis to control the size and form of upconverting AYF_4_:Yb, Er (with A 14 Na, Li) NCs showed that altering the period of NC formation allows one to adjust the size of the nanoparticles. The concentration and content of reactants can be used to affect the shape of the resultant NCs [54]. Modlitbová et al. [78] investigated the effects of 3 different sizes of photon-UCNPs on the model plant *Zea mays*. A recent study also confirmed that small-size UCNPs show a much higher uptake capability than other UCNPs [79]. A recent study by Chen et al. [80] confirmed that small-size UCNPs show a much higher uptake capability than other UCNPs. They used endothelial cells (ECs) to study the potential toxicity of 3 different sizes of europium-doped NaYF_4_ (NaYF_4_:Eu^3+^) UCNP. The results demonstrated that these UCNPs may enter ECs and, in a size-dependent manner, decrease cell viability, trigger the release of intracellular lactate dehydrogenase (LDH), increase ROS levels, and lower cell mitochondrial membrane potential (MMP). In addition, the activation of caspase-3 causes the cells to undergo apoptosis; intercellular cell adhesion molecule-1 (ICAM1) and vascular cell adhesion molecule 1 (VCAM1), 2 genes that are associated with inflammation, are expressed more frequently in larger sizes than in smaller ones. The surface charge of UCNPs, determined by the presence of surface functional groups or coatings, can affect their interactions with biological molecules and cells [80].

Kembuan et al. [65] investigated the effect of different sizes of silica coatings and found that the cytotoxicity can be reduced by increasing the size of UCNPs. This study found that the thickness of UCNPs is indirectly proportional to cytotoxicity; that is, thicker silica coatings are less toxic to macrophages than UCNPs with thinner silica coatings. Rafique et al. [81] proposed a simple procedure for producing hydrophilic UCNPs in various sizes and morphologies. They thoroughly investigated various experimental conditions, including the amounts of reactants and NaF, dopant concentrations, and hydrothermal reaction durations. According to their findings, shape, phase evolution, uniformity, and increased UC luminous intensity were caused by high reactant and NaF contents, suitable dopant concentrations, reaction duration, and reaction time. In addition, the as-prepared highly luminous UCNP displayed low cytotoxicity (>80%) in HeLa cells, even at a dose of 1,000 μg/ml. The improved UCNPs were also suitable for in vitro live cell imaging.

### Surface charge

The surface charge of UCNPs, determined by the presence of surface functional groups or coatings, can affect their interactions with biological molecules and cells. Positively charged UCNPs may have higher cellular uptake and potential cytotoxic effects than negatively charged or neutral UCNPs [64]. Although imaging, gene transfer, and drug delivery appear to be more effective in the presence of a positive charge, the cytotoxicity of such constrictions is more significant. The optical properties of UCNPs, enabling them to produce higher-energy photons while absorbing lower-energy ones, are particularly fascinating. The cytotoxicity and bioaccumulation potential of UCNPs is closely linked to their optical properties, such as emission spectra, reactive oxygen species (ROS) production, necrosis, and inflammation. Their therapeutic efficacy and potential toxicity may be affected by light output in specific wavelengths (UV, visible, or NIR), especially when heat or ROS are generated. Moreover, the interactions of UCNPs with biological systems are significantly affected by size, surface charge, and functionalization. It is essential to understand and optimize their optical characteristics to reduce cytotoxicity and enhance the therapeutic and diagnostic advantages of UCNPs [82].

To better understand the relationship between cytotoxicity, internalization, and subcellular localization in normal and cancer cell lines, Li et al. [83] synthesized UCNPs with various surface charges (positive, negative, and neutral). UCNPs with negative charges are primarily internalized within cancer cell lines, whereas a broader range of the investigated cell lines take up those with positive or neutral charges. This pattern highlights the distinct behaviors of UCNPs based on their surface charges, which influence their internalization across different cell lines. Moreover, it was demonstrated that surface charges also influence the localization of these UCNPs in different cell compartments, such as the cytoplasm, mitochondria, and lysosomes, and the cytotoxicity of these charged UCNPs, in turn, significantly depends on the localization regions.

Arellano et al. [84] synthesized mono-dispersed UCNPs with 4 different types of surface coatings and studied their impact on cell cytotoxicity and endocytosis/exocytosis. The results showed that PEGylation, while effective for colloidal stability purposes, inhibited extensive cell internalization, whereas polymer coating of UCNPs using poly (isobutylene-alt-maleic anhydride) constituted an outstanding design approach for their subsequent biomedical applications. However, small ligand-based coatings are insufficient and frequently cause partial particle aggregation. Similarly, Jin et al. [85] used a hydrothermal technique to design UCNPs with polyvinylpyrrolidone coatings (UCNP-PVP). Next, they used polyethyleneimine (PEI) and poly (acrylic acid) (PAA) to perform a ligand exchange reaction on UCNP-PVP to produce UCNP-PEI and UCNP-PAA, respectively. These polymer-coated UCNPs showed suitable aqueous medium and crystal phase dispersibility, shared a similar TEM and dynamic light scattering size distribution, and displayed similar upconversion luminescence efficiency. However, compared to its neutral or negative equivalents, the positively charged UCNP-PEI demonstrated significantly increased cellular absorption.

### Aggregation and stability

The tendency of UCNPs to aggregate or agglomerate can influence their toxicity. Aggregated UCNPs may have altered physicochemical properties and cellular interactions, which can lead to different biological responses compared to well-dispersed UCNPs [68]. The retention and cellular uptake of inorganic nanoparticles in tumors can be improved through the controlled aggregation of nanoparticles, and their modification with a mixed-charge zwitterionic surface can simplify the process of obtaining both pH sensitivity and stealth properties [86]. The stability of UCNPs in biological environments also affects their behavior and potential toxicity [5,63]. It is essential to consider these factors collectively to evaluate the toxicity of UCNPs comprehensively and to make informed decisions regarding their safe and responsible use in biomedical applications. The biocompatibility of UCNPs can be improved by techniques such as surface modification, and size and shape optimization. Additionally, cautious administration strategies and in vivo monitoring can guarantee that UCNPs deliver the desired effects without inadvertently damaging healthy tissues. UCNPs can be efficiently used for a variety of biological applications, such as imaging, drug delivery, and therapeutic interventions, by addressing these issues [87].

## Administration Routes for UCNPs and their ADME Process and Kinetics

The choice of administration route of UCNPs significantly influences their biodistribution, metabolism, and excretion, as well as their toxicity in the body. The different administration routes for UCNPs include intravenous (i.v.) and, intraperitoneal (i.p.) injections, intragastric (i.g.) administration, and inhalation in the form of aerosols. Each route has different effects on toxicity and behavior within the body, depending on aspects such as particle size, surface properties, composition, and dosage. Several studies have shown that UCNPs may induce oxidative stress, inflammation, and cytotoxicity based on these characteristics and specific exposure routes. Biodistribution and bioavailability of UCNPs within the body mainly rely on the mode of exposure. The i.v. injection involves directly introducing UCNPs into the bloodstream, allowing for rapid systemic distribution, mainly in organs such as the liver. This is the most common route used in biomedical applications, such as targeted imaging and drug delivery. The i.p. injection involved the dose of UCNPs into the peritoneal cavity, allowing efficient absorption into the systemic circulation. In contrast, the i.g. administration is the oral ingestion of UCNPs, which is the most convenient and noninvasive method for delivering UCNPs. Yu et al. [88] investigated the toxicity, biodistribution, and excretion pattern of PEI-modified NaYF_4_:Yb, Er UCNPs in mice through 3 different routes: i.p., i.v., and i.g. According to these findings, the distribution patterns of PEI@UCNPs differed depending on the method of administration. After i.p. administration, accumulation was observed in the reticuloendothelial system primarily in the spleen and slightly in the liver, and small leakage in the duodenum was recorded 30 days after injection. Conversely, after i.v. administration, accumulation was found predominantly in the spleen and liver, whereas a minimal amount of UCNPs was observed in other organs. This shows that the spleen is the target organ for UCNPs accumulation via the i.p. and the i.v. routes. However, i.g. administration led to primary accumulation in the small intestine within an hour, which then gradually decreased within 48 h. The biodistribution of PEI@UCNPs was also examined using positron emission tomography (PET) imaging after intravenously injecting mice with ^64^Cu-NOTA-PEI@UCNPs. Results showed that radioactive signals were mostly detected in the liver and lungs within 0.5 to 2 h, possibly due to accumulation of nanomaterials in the lung and mononuclear phagocytic system of the liver, whereas no signals were detected in other organs. Moreover, the excretion patterns of PEI@UCNPs differed depending on the route of administration. After i.p. administration, PEI@UCNPs were slowly excreted via urine and feces over 30 days. However, after i.v. and i.g. administration, the excretion occurs via feces over 48 and 24 h, respectively [88]. Thus, understanding the potential toxicity of UCNPs and their responses to different routes of action is crucial for their safe and effective utilization in biomedical applications. Gao et al. [89] investigated the i.p. administration of citrate-modified UCNPs (cit-UCNPs) comprising NaLuF_4_:Yb, Tm@NaLuF4, and citrates into male mice. The results showed accumulation of cit-UCNPs within the regional and local tissues of the abdominal cavity. It was observed that i.p. administration resulted in higher tumor-targeting efficiency and faster clearance, hence enhancing the contrast-enhanced imaging for pancreatic cancer diagnosis and monitoring. Yuan et al. [90] also highlighted the potential of utilizing biointerface-camouflaged UCNPs and the i.p. route for improving the accuracy and efficiency of contrast-enhanced imaging in pancreatic cancer diagnosis and monitoring. Oral delivery of drugs is a common method in scientific research with small animals such as mice, but it is poorly absorbed due to its larger size than traditional medicine [91]. As a result, it is critical to investigate whether these nanoparticles can pass through epithelial barriers, particularly the intestinal barrier. Limited data are available on the bioavailability of these nanoparticles following oral exposure [92]. The bioavailability, biodistribution, and toxicity of orally administered NaYF_4_:Yb, Er@SiO_2_ nanoparticles, with an average diameter of 50 nm, were studied. Upon oral administration of NaYF_4_:Yb, Er@SiO_2_, nanoparticles mainly accumulated in the bone, stomach, and colon. Conversely, when administered intravenously, these nanoparticles are mainly localized in the liver and spleen [5].

Xiong et al. [66] studied the biodistribution and toxicity of NaYF_4_:Yb^3+^, Tm^3+^ coated with PAA in mice. Initially, after i.v. administration, UCNPs were removed from the bloodstream, but were found in the spleen, liver, and, to a lesser extent, lungs. Accumulation of UCNPs occurred in the spleen within 24 h, and UCNPs were gradually degraded in the liver. A weak signal was detected in the heart and kidneys. The liver and spleen are crucial organs for removal of UCNPs from the body. Even after 2 weeks, the emission signal was still detectable in both organs. Three months after injection, the intestinal system continued to emit UCNP signals [66]. Cheng et al. [77] conducted an extensive in vivo investigation of β-NaYF4:Tm^3+^, Yb^3+^ UCNPs coated with PAA and PEG. This study found no evidence of organ damage or lesions in mice when intravenously administered at a dose of 20 mg/kg. Additionally, no abnormalities were observed in the hepatic enzyme levels or serum biochemistry. Interestingly, TEM imaging and ion-coupled plasma (ICP) quantification revealed aggregates of nanoparticles in the liver 7 days after injection [77]. According to Xing et al. [93], when NaYbF_4_ UCNPs were administered at a dose of 150 mg/kg to mice, the maximum accumulation of nanoparticles is observed at 0.5 and 24 h in the spleen and liver. After 24 h, nanoparticles accumulated at low concentrations in the kidneys and lungs. Excretion was observed 7 days after injection through urine and feces. After 30 days of administration, no UCNPs were detected in these organs. The results also demonstrate that there are no toxic effects on any organ due to the prolonged distribution time of PEI UCNPs [93]. Recently, Machová Urdzíková et al. [94] synthesized the hexagonal NaYF_4_:Yb, Er UCNPs with sizes of 25 nm (S-UCNPs) and 120 nm (L-UCNPs). They conducted an experiment using rat mesenchymal stem cells (rMSCs) and C6 cancer cells. The results showed that both large and small UCNPs were internalized in the lumen of endosomes after intravenous injection and eliminated through the body via the hepatobiliary route after 96 h. L-UCNPs caused oxidative damage to rMSCs, whereas no significant difference was observed in C6 cells [94].

## Toxicity Assessment of UCNPs

The properties and performance of nanomaterials can be altered, which has led to their widespread application in technology and consumable products relevant to everyday life. Given the growing prevalence of nanomaterial applications, evaluating their toxicity must be the primary step in establishing safety protocols for their management and disposal [95].

UCNPs have significant potential for biomedical applications, such as magnetic resonance imaging (MRI), fluorescence imaging, drug delivery, cancer detection, and treatment. Over the past decade, the use of UCNPs as imaging agents in the NIR optical window of biological tissues has encouraged extensive research in the field of nanomedicine. A comprehensive assessment of their functionalization, nanotoxicological impact at the molecular and cellular levels, and biocompatibility with organisms is crucial for biomedical applications. UCNPs can generate ROS that cause DNA damage, affect cell growth via protein oxidation, and impact mitochondrial respiration [96]. The toxicity of UCNPs varies according to their size, shape, surface chemistry, dose, and other characteristics. Surface coatings can significantly reduce potential toxicity by enhancing stability and limiting interactions with biological components. Coating the UCNPs with a hydrophilic ligand or an extra hydrophilic layer is typically required for surface modification [6,78,79]. Numerous methods have been used to reduce UCNP toxicity. These include surface modification with biocompatible coatings, regulation of UCNP size and shape, utilization of biodegradable materials, selection of inert and safe core materials, thorough toxicity assessments, and monitoring of dosage and exposure. Combining these strategies can improve the biocompatibility and safety profile of UCNPs, thereby enabling their secure implementation in biomedical applications. The incorporation of surface coatings plays a crucial role in modulating the toxicity and biocompatibility of nanoparticles. The choice of surface coating significantly influences the toxicity and biocompatibility of nanoparticles. This knowledge is crucial for optimizing nanoparticle design for therapeutic applications while minimizing potential risks associated with their use in clinical settings [24].

A comprehensive analysis of various coating materials for UCNPs reveals significant differences in their toxicity reduction and biocompatibility. Table 4 summarizes quantitative data from relevant studies comparing different coatings such as PEG, silica, citrate, and others regarding their impact on cytotoxicity and overall biocompatibility.

**Table 4.** A comparative table of surface coatings on UCNPs cytotoxicity and biocompatibility

Coating material	IC_50_ (μg/ml)	Cytotoxicity observations	Biocompatibility	References
Uncoated UCNPs	98.5–774.6	High cytotoxicity; significant decrease in cell viability	Low	[5]
Citrate-coated UCNPs	563.4 (20 nm)	Reduced cytotoxicity; lower fluoride release	Moderate	[5]
EDTMP-coated UCNPs	No cytotoxic effects	Excellent biocompatibility; minimal ion leakage	High	[5]
PMAO-coated UCNPs	Not specified	Noticeable cytotoxicity; morphological changes in treated cells	Moderate	[134]
Silica-coated UCNPs (thick)	Not specified	Lower toxicity; stability increases with shell thickness	High	[65]
Silica-coated UCNPs (thin)	Not specified	Higher cytotoxicity compared to thicker shells	Moderate	[65]
PEG-coated UCNPs	Not specified	Moderate stability; potential deformation affecting luminescence	Moderate	[11]
Alendronate-coated UCNPs	Not specified	Most cytotoxic due to inherent toxicity of alendronate	Low	[11]
PDMA-coated UCNPs	Not specified	High cellular uptake and low toxicity, suitable for cancer therapy	High	[135,136]
PMAO-coated UCNPs	Not specified	Low toxicity; good colloidal stability	High	[11]
PEG-oleate bilayer UCNPs	Significant toxicity	Toxicity due to ligand dissociation exposing hydrophobic cores	Low	[109]

Many toxicological studies have been performed on cells and organs, both in vivo and in vitro, to investigate the potential toxic effects of UCNPs. Every experimental model has distinct benefits. However, several limitations exist. In vitro cell culture models are helpful for preliminary tests, although they lack organismal complexity. On the other hand, in vivo animal models offer a more thorough understanding of systemic toxicity but can be expensive and present ethical problems.

The properties and performances of nanomaterials can be altered, which has led to their widespread application in technology and consumable products relevant to everyday life. Given the growing prevalence of nanomaterial applications, evaluating their toxicity must be the primary step in establishing safety protocols for their use, management, and disposal. Hondroulis et al. [97] effectively developed an electrical impedance sensing (EIS) chip and assessed its applicability for diverse cytotoxicity evaluations using electrochemical experiments. The results demonstrated that the EIS chip displayed a consistent and predictable response when assessing the effects of different nanomaterials-AuNPs (10 and 100 nm), AgNPs (10 and 100 nm), SWCNTs (cut and uncut), and CdO-on CCL-153 and RTgill-W1 cells. Furthermore, the EIS chip exhibited sufficient sensitivity to quantify a cell’s micro-motion, enabling the observation of cytotoxicity development and illustrating the kinetic effects of nanoparticles on the cells. Moreover, the EIS chip facilitated swift, real-time, and multisample analysis, establishing a versatile, noninvasive instrument capable of delivering quantitative data regarding changes in cellular function under diverse nanomaterial exposures [97].

Shah et al. [98] investigated nanotoxicity evaluation on an individual cell and a minor cell population utilizing electrochemical impedance spectroscopy and a microelectromechanical system (MEMS) device. They exhibited a regulated capture of PC12 cells in microwells of varying dimensions (to accommodate variable cell quantities) employing a synergistic approach of surface functionalization and dielectrophoresis. The current method offers a swift nanotoxicity response, in contrast to previous traditional methods. This study was the first to illustrate the comparative response of a single cell and small cell colonies on the same MEMS substrate when exposed to metal oxide nanoparticles. They established that the cellular microenvironment influences cell behavior and reactions to nanomaterials. The findings of this experimental work propose a novel idea to investigate the role of cellular communication in the dissemination of toxicity within a cell population [98].

UCNPs have several intriguing properties, including strong photostability, reduced autofluorescence background, crisp emission bandwidths, excellent chemical/physical stability, and good biocompatibility. With proper control over the synthesis circumstances (e.g., chemical ratio, reaction temperature, and heating up speed) and the composition of host and dopant metal ions, several UCNP characteristic parameters (such as size and form) can be altered [99]. Numerous methods, including salinization, ligand interactions, ligand exchange, and chemical reactions of surface ligands, have been documented for surface modification of UCNPs. The blood–brain barrier (BBB) penetration of UCNPs is significantly influenced by their form and surface modification [100]. Unquestionably, various applications that take advantage of lanthanide upconverting nanoparticles’ advantages in vitro are still very promising and competitive with more conventional methods. Future studies on UCNPs for biomedical applications are encouraged by the numerous reports of lanthanide-doped UCNPs’ negligible or low toxicity.

**Fig. 6.**
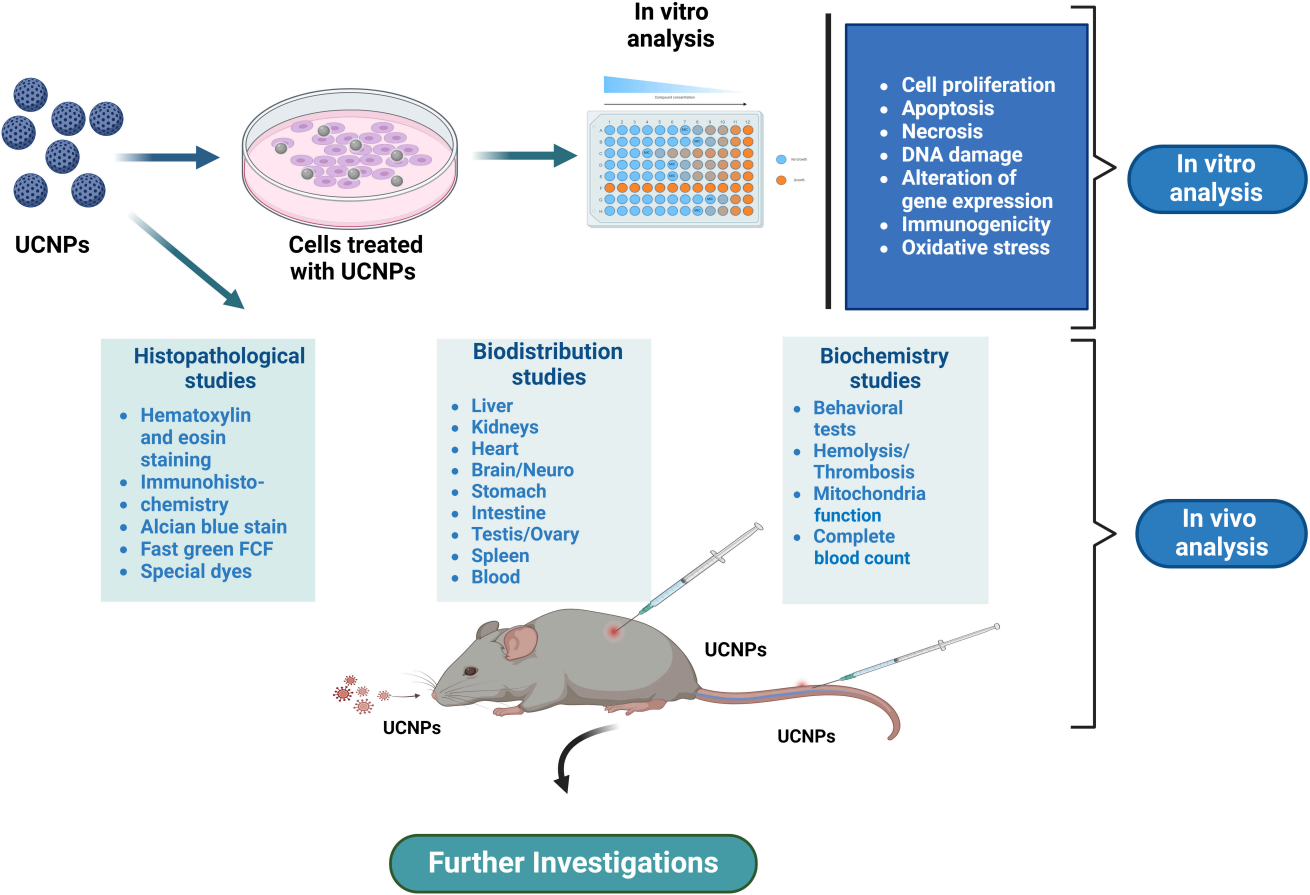
Diagrammatic representation of toxicity assessment of upconversion nanoparticles via in vitro and in vivo studies showing the details of assays that can be performed in preclinical studies.

Figure 6 shows a schematic representation of the steps involved in both in vitro and in vivo assays and approaches for the toxicological assessment of nanoparticles. For the in vitro analysis, the desired cell lines were treated with UCNPs to assess their cellular and molecular toxicity. For biochemical, histopathological, and biodistribution studies, in vivo analysis was performed in healthy mice by injecting UCNPs through i.v., intramuscular (i.m.), and i.g. routes.

### In vitro toxicity of UCNP

**Fig. 7.**
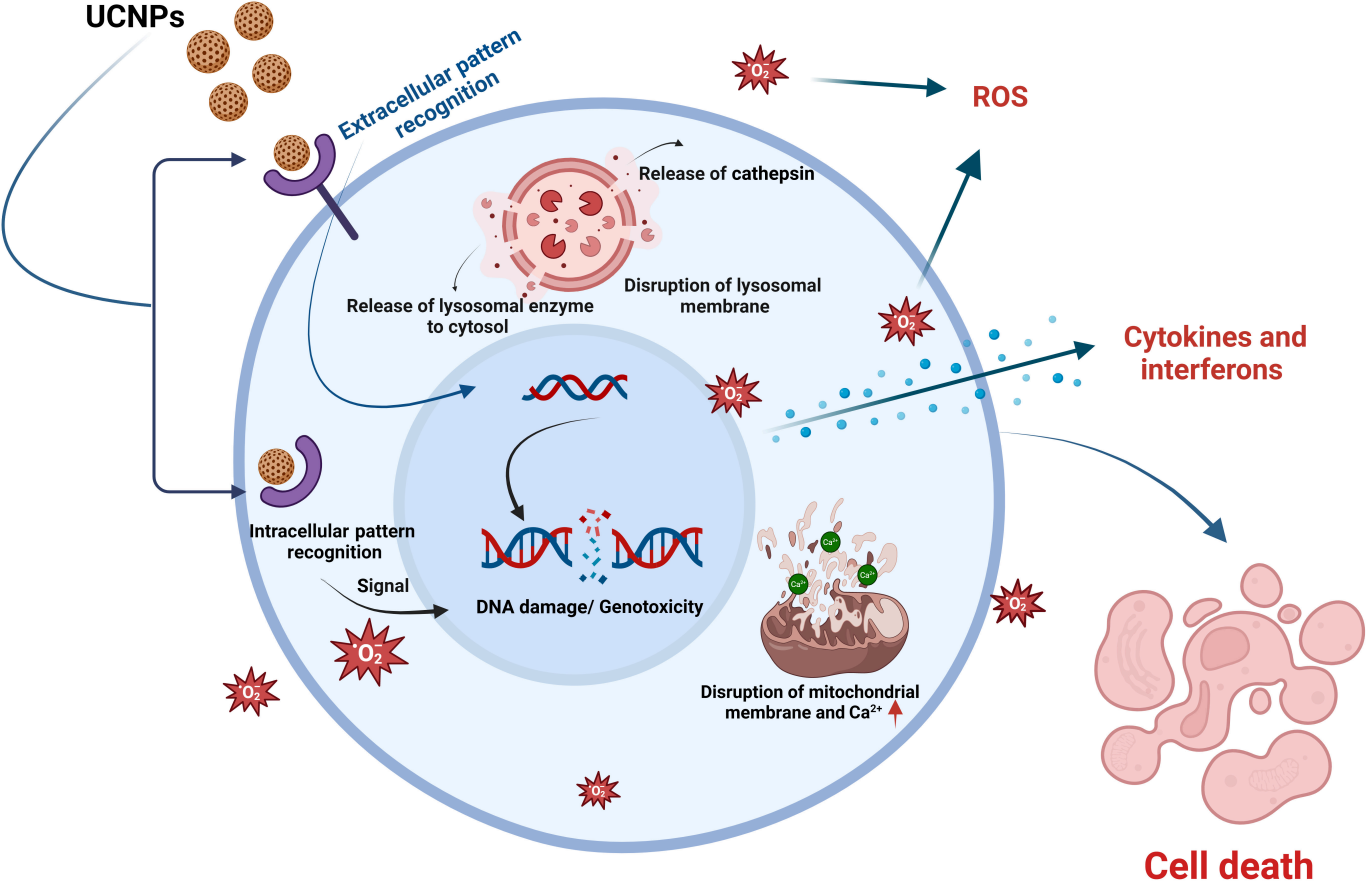
Molecular mechanism of toxicity of upconversion nanoparticles in a living system.

In vitro toxicity studies of UCNPs typically involve investigating variables such as cellular uptake, cytotoxicity, cell viability, genotoxicity, ROS production, and inflammatory reactions (Fig. 7). These studies have facilitated the development of safe and efficient applications in numerous areas of biology and medicine by providing insightful information on the biocompatibility and potential toxicity of UCNPs. Several UCNPs have been investigated for cytotoxicity in vitro. Cordonnier et al. [101] synthesized NaYF_4_:Yb, Tm@NaYF_4_ core/shell UCNPs coated with oleic acid. PEG ligands with dissimilar anchoring groups (phosphate, bis-, and tetra-phosphonate-based) were used to hydrophilize the UCNPs, and a tetra-phosphonate PEG (2000) ligand with sustained colloidal stability was selected. The cytotoxicity of prostate-specific membrane antigen-targeting ligands (glutamate–urea–lysine derivatives, KuEs) or radiolabeled UCNP@KuE particles coated with 10% or 100% surface density of KuE was evaluated in prostate cancer cell lines (LNCaP-Luc and PC3-Luc) and human fibroblasts. It was observed that the UCNP@KuE particles did not cause significant toxicity to the cells during a 24-h incubation period and at tested concentrations of 1.2 μg/ml to 1.7 mg/ml. Moreover, flow cytometry and competitive binding assay experiments confirmed the good affinity of UCNP@KuE particles toward prostate-specific membrane antigen (PSMA)-positive LNCaP-Luc cells compared to nontargeted UCNP@CO_2_H particles [101]. A successful delivery platform based on UCNPs that targets the delivery of miRNA provides a novel approach to the field of bioengineering and nanotechnology for tumor therapy. Functionalized NaYF_4_:Yb/Tm UCNPs synthesized and coated with SiO_2_ exhibited sound upconversion emission and uniformity. UCNPs carrying miR-145 demonstrate exceptional biocompatibility, high cellular uptake, and increased expression of miR-145, leading to significant arrest at the G1 phase of cell cycle and downregulation of cell cycle proteins CCND1, CDK6, and CCNE2 [102].

The dose-dependent cytotoxicity of NaYF_4_: Yb, Er UCNPs was examined in HepG2 cells, with an IC_50_ value of 100 μg/ml. The authors found that UCNPs caused cell death mainly by producing ROS, which can oxidize and damage lipids, proteins, and DNA and are toxic to cells. Additionally, HepG2 cells internalize UCNPs through endocytosis, and lysosomes break down the internalized UCNPs [103]. In a study by Chen et al. [80], the size-dependent cytotoxicity of NaYF_4_:Eu^3+^ nanoparticles was evaluated using MTT and LDH assays in human umbilical vein endothelial cells, with larger particles being more toxic than smaller ones. UCNPs of 3 different diameters (50, 150, and 350 nm) were tested at 20, 40, 60, 80, and 100 μg/ml concentrations. These findings revealed that these nanoparticles may enter endothelial cells in a size-dependent manner, decrease cell survival, trigger the release of intracellular LDH, elevate ROS levels, and reduce cell MMP levels. Additionally, ROS have been suggested to be the mechanism by which NaYF_4_:Eu^3+^ nanoparticles cause cell death, and the activation of caspase-3 causes the cells to undergo apoptosis, with the up-regulation of inflammation-related genes (ICAM1 and VCAM1) [80].

Several UCNPs have been tested for their cytotoxicity in vitro and have shown low cytotoxicity at concentrations up to 100 μg/ml, with an IC_50_ value of 200 μg/ml [12,35,104–108]. Das et al. [109] investigated the toxic effects of functionalized UCNPs: PEG-NPs, oleate ligands-NPs, and bilayer PEG-oleate-NPs. Due to the functionalization of UCNPs, significant toxicity was recorded in propidium iodide viability and calcein assays [109]. Zhu et al. [74] reported that UCNPs-Ce6-mediated PDT led to decreased cell viability with increasing nanoparticle concentrations from 2 to 16 μg/ml when exposed to constant laser irradiation for 60 s. Cell viability also reduced with increasing exposure time to laser irradiation when incubated at a constant concentration of 16 μg/ml [74] UCNPs-Ce6. The above results suggest that cytotoxic effects are enhanced with both increased UCNPs concentration and increased laser exposure time [74]. Mishchenko et al. [110] examined the cytotoxicity of UCNPs on glioma and primary hippocampus cells. UCNPs were toxic to the primary hippocampus and glioma cells at 100 μg/ml doses. It demonstrated mild cytotoxicity in glioma cells at concentrations of 25 and 50 μg/ml. It was envisaged that while UCNPs can be moderately cytotoxic to glioma cells, they are not hazardous to normal brain cells. The authors also proposed that the cytotoxicity of UCNPs in glioma cells might result from ROS production [110]. Kembuan et al. [65] examined the cytotoxicity of different-sized silica-coated UCNPs in RAW264.7 macrophages. The researchers found that UCNPs with thicker silica coatings were less toxic than thinner silica coatings to RAW 264.7 cells. Silica coating protects the UCNPs from degradation by the cells, which reduces the discharge of toxic lanthanide ions from the UCNPs [65]. In a recent study by Bietar et al. [111], the toxicity of square bipyramidal UCNPs containing a LiYF_4_:Yb^3+^, Tm^3+^ core and 2 different silica coating surfaces (Si-UCNPs and AzSi-UCNPs) was investigated. Rigorous analysis was performed using a series of stress biomarkers in fibroblasts and renal proximal tubule cells to evaluate the toxicity of UCNPs. These results demonstrate that short-term exposure to UCNPs has no significant effect on cell size and viability. Surface modification with silica coating has a diminished effect on cells after an incubation period of 24 h [111].

### Molecular mechanisms of UCNPs’ toxicity

The physicochemical characteristics (such as size and surface charge) of UCNPs play a role in their complicated toxicity within the biological cells. To create safer nanoparticles and maximize their use in biomedicine, it is crucial to comprehend the exact molecular processes by which UCNPs cause toxicity. However, due to the different physical and chemical characteristics of UCNPs compared to other forms of nanoparticles, it is practically impossible to compare their nanotoxicity directly. Furthermore, UCNPs with varying hydrodynamic sizes, shapes, compositions, charges, and surface functional groups have distinct absorption and clearance rates and mechanisms that depend on the particular cell or tissue [5,72,112]. Some of the most commonly comprehended toxicity mechanisms of UCNPs are as follows**:**

#### ROS production

Excess ROS production is considered the primary mechanism of action of UCNPs. UCNPs generate extra ROS upon excitation or through interactions with biological molecules, which induces oxidative stress by disrupting the cellular redox balance; damaging biomolecules such as lipids, proteins, and DNA; and triggering inflammatory responses (Fig. 7). NP treatment disturbs the redox homeostasis and causes inflammation at an early stage, moreover reaching the stage of cell death. Cellular damage upon exposure to UCNPs is the result of a reduction in the amount of antioxidants such as glutathione, production of lipid peroxide, and ROS [28]. A few studies have reported ROS-mediated damage to living cells in animal models, including mouse and zebrafish embryos [35,113–115]. UCNPs induce cell death and apoptosis through ROS generation [116,117]. In some cells, ROS manifest as phototoxicity in the presence of light [118]. Chen et al. [80] reported that UCNPs taken up by endothelial cells decrease cell viability, trigger intracellular LDH release, increase ROS production, and decrease MMP in a dose- and size-dependent manner. Wang et al. [103] validated that UCNPs can cause cytotoxicity to HepG2 cells in a dose- and time-dependent manner, which is probably facilitated by oxidative stress and ROS generation. When cells were treated with 200 mg/l UCNPs for 48 h, apoptosis and inflammation were induced, ROS were generated, and other metabolic processes were disrupted. ICP-MS analysis revealed that cytotoxicity is dependent on the surface properties of UCNPs rather than their tested sizes (35 and 55 nm) [103].

#### Inflammatory response

UCNPs have emerged as a promising platform for modulating immune responses, particularly through their influence on cytokine cascades such as interleukin-6 (IL-6) and tumor necrosis factor-α (TNF-α). Their unique optical properties allow deep tissue penetration and targeted delivery of antigens and adjuvants, which can significantly enhance immune activation. UCNPs utilize a nonlinear optical process where low-energy NIR photons are absorbed and converted into higher-energy UV or visible light. This property minimizes light scattering in biological tissues, facilitating effective delivery and monitoring of therapeutic agents [28,114,115,119].

**Fig. 8.**
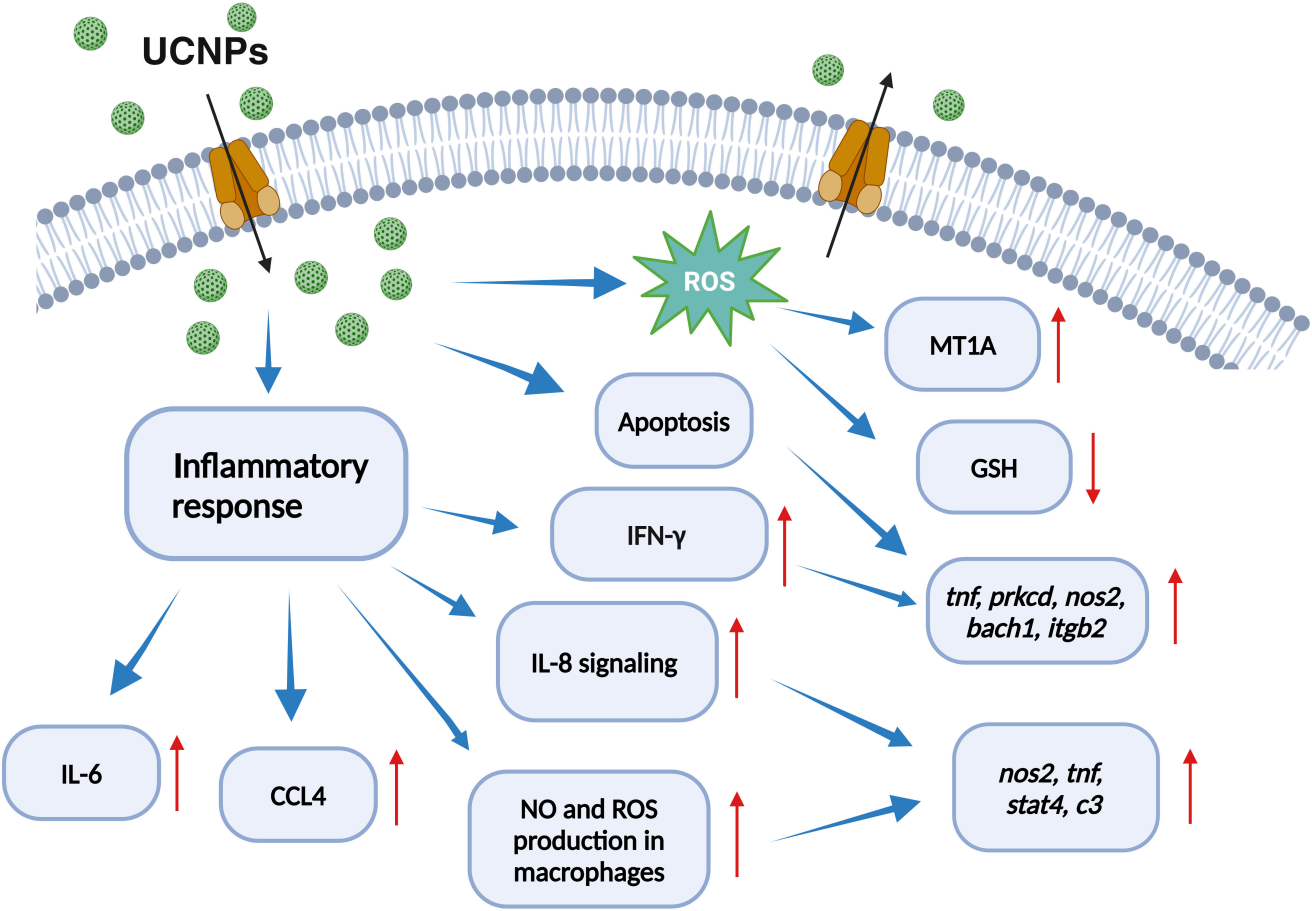
Upconversion nanoparticle inflammatory response pathways. The pathways include inflammatory response cytokine signaling (e.g., interleukin-6 [IL-6], tumor necrosis factor-alpha [TNF-α], and interferon-gamma [IFN-γ]), reactive oxygen species (ROS) production, and apoptosis. Additionally, the figure highlights the activation of macrophages, leading to nitric oxide (NO) and ROS production, and the roles of key genes and proteins such as nitric oxide synthase 2 (NOS2), protein kinase C delta (PRKCD), and signal transducer and activator of transcription 4 (STAT4).

Upon interaction with immune cells, particularly dendritic cells (DCs), UCNPs can stimulate the production of proinflammatory cytokines. For instance, studies have shown that the presence of UCNPs linked with specific antigens and adjuvants can lead to increased secretion of IL-6 and TNF-α, which are crucial for initiating and propagating immune responses [113–116,119,120]. The degree of cytokine release is often dose-dependent, suggesting that careful modulation of UCNP concentration can optimize immune activation while minimizing potential adverse effects [106,121,122]. Figure 8 shows UCNP inflammatory response pathways.

UCNPs can be engineered to deliver antigens directly to DCs, enhancing their maturation and ability to present antigens to T-cells. This process is vital for eliciting robust and adaptive immune responses. The coupling of UCNPs with immunostimulatory agents like Pam3CSK4, has been shown to further amplify this effect, resulting in higher levels of cytokines, such as IL-6 and TNF-α [28,97,114,116,117,122]. The activation of DCs by UCNPs leads to the secretion of various cytokines that orchestrate the immune response. For example, TNF-α plays a pivotal role in inflammation and the activation of other immune cells, while IL-6 is involved in both proinflammatory and anti-inflammatory responses. The balance between these cytokines can dictate the outcome of the immune response, influencing whether it is protective or pathogenic [28,106,116].

Recent studies involving UCNPs have explored their role in modulating cytokine cascades, particularly in cancer therapy and immunotherapy. Evidence of NPs-Nd_2_O_3_-induced inflammation in the lungs of Sprague-Dawley rats has been reported by Kim et al. [123]. The rats were given NPs-Nd_2_O_3_ treatment through inhalation at a concentration of 0.5 mg/m^3^ for 28 days with a repeat cycle of 5 days per week. Pulmonary inflammation was also observed in rats treated with NPs-Nd_2_O_3_. The inflammatory response is mediated by increased levels of inflammatory cytokines and chemokines. Inflammation is accompanied by damage to the lung tissue, including increased lung weight and alveolar proteinosis. The authors concluded that NPs-Nd_2_O_3_ can cause lung inflammation and damage. Chan and Hsiao et al. [121] demonstrated similar results, which confirmed that UCNPs induced the release of ROS, inflammatory chemokines, and cytokines, leading to the recruitment of neutrophils and macrophages to the lungs. UCNPs also activate the complement system, which leads to lysis of lung epithelial cells.

In a study focusing on the combination of UCNPs with PDT and immunotherapy, researchers observed significant increases in immune-related cytokines, such as IL-12p40, interferon-γ (IFN-γ), and TNF-α, after treatment with UCNPs-Ce6-R8372. This combination not only enhanced antitumor effects but also activated tumor-specific immune responses [117,121,124]. Another study demonstrated how UCNPs can be used to control the release of CpG oligonucleotides (CpG ODNs), which are potent immunotherapeutic agents. Under NIR light irradiation, the UV light generated by UCNPs breaks photocleavable bonds releasing CpG ODNs. These molecules bind to Toll-like receptor 9 (TLR9), promoting DC maturation and inflammatory cytokine production [121,125]. UCNPs have been explored for enhancing DC-based vaccines by conjugating antigens like ovalbumin onto their surface. This approach results in efficient engulfment by DCs, leading to their maturation and enhanced antigen-specific T-cell responses, including increased IFN-γ production [28,121]. The ability of UCNPs to modulate cytokine responses has implications for immunotherapy, particularly in cancer treatment. By enhancing the presentation of tumor antigens and stimulating a favorable cytokine profile, UCNPs could improve the efficacy of cancer vaccines and other immunotherapeutic strategies [124,126]. Additionally, their low toxicity profile makes them suitable candidates for clinical applications where precise control over immune modulation is necessary [28,106,122,126]. Thus, UCNPs represent a versatile tool in immunotherapy by effectively influencing cytokine cascades like IL-6 and TNF-α. Their capacity to enhance DC activation and modulate immune responses positions them as valuable agents in the development of advanced therapeutic strategies.

#### Apoptosis/necrosis

UCNPs have numerous effects on cells due to apoptosis or necrosis, such as cell differentiation and proliferation, cell cycle regulation, cell death, and DNA damage (Fig. 7). These effects depend on nanoparticle size, type, shape, surface charge, and functionalization. Therefore, toxicity assessment of UCNPs is a complex process that requires multiple factors to address and interpret results correctly. However, a systemic and standard approach has yet to be established [122]. Ge et al. [127] have reported that necrosis is responsible for increased ROS production and leads to mitochondrial damage. In this study, NaYF_4_:Eu^3+^ was shown to increase ROS levels and decrease mitochondrial membrane proteins, which may damage DNA and ultimately affect cell cycle progression [127]. According to one study, using UCNPs-Ce6 in PDT can lead to cellular damage and apoptosis in THP1 cells via ROS outbursts and lipid peroxidation pathways, leading to proteotoxicity [74]. Nd_2_O_3_ NPs cause toxicity and cerebrovascular abnormalities in zebrafish embryos through the apoptotic pathway [128]. At high doses (>200 μg/ml), Nd_2_O_3_ NPs disrupt embryonic development. Morphological observations showed a dose-dependent increase in mortality and malformation rates. At 120 hours post-fertilization (hpf), the median lethal concentration (LD_50_) of Nd_2_O_3_ NPs was 203.4 μg/ml. Nd_2_O_3_ NP-treated embryos also displayed decreased heart rate and significant arrhythmia. A significant decrease in cerebrovascular effects was observed at intermediate concentrations (100 and 200 μg/ml). Reduced brain blood vessel autophagic flux and elevated neuronal apoptosis may affect vessel sprouting and account for the disappearance of cerebrovascular accidents.

### In vivo toxicity of UCNPs

**Fig. 9.**
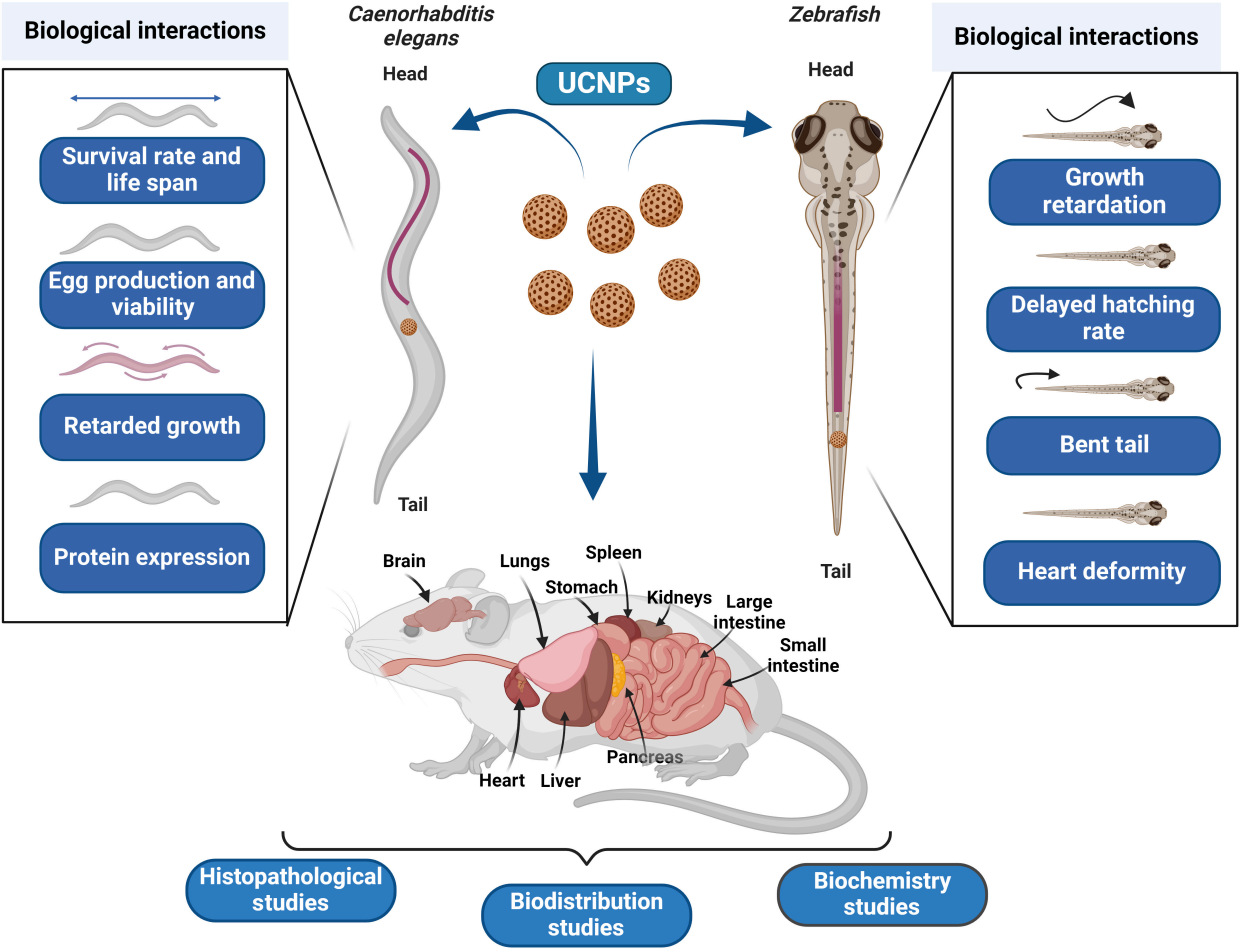
Interaction of upconversion nanoparticles with living systems.

Selection of animal models is the first stage of in vivo experiments. Despite the development of numerous animal models, a standard and reliable model is lacking. Zebrafish, rabbits, and *Caenorhabditis elegans* have been used in nanotoxicological experiments, in addition to the most typical experimental mice and rats (Fig. 9). Here, we summarize the recent advancements in the toxicological assessment of UCNPs from the standpoint of living systems.

The utilization of UCNPs from rare-earth fluoride NCs for bioimaging with minimal or no toxicity has garnered significant interest, particularly for Yb^3+^/Tm^3+^-doped NCs [108,129,130]. Abdul Jalil et al. and Zhang [108] assessed the cytotoxicity of silica-coated NaYF_4_:Yb by exposing rat skeletal myoblasts and bone marrow-derived mesenchymal stem cells to these NCs. These findings highlight that the biocompatibility of the NCs is satisfactory both in vivo and in vitro [108]. Cheng et al. [77] studied the biodistribution and biotoxicity of Ln-UCNPs after coating with PAA in mice and did not observe any significant toxic effects on the liver or spleen. Zhou et al. [107] assessed the bioimaging and toxicity of NaYF_4_:Yb, Tm NCs in *C. elegans*. In vivo studies were performed on *C. elegans* treated with 100 μg/ml NC. The results were analyzed using the following criteria: GFP expression, lifespan, growth rate, egg production, viability of worms, and ingestion and excretion of NCs from worms. The results showed negligible toxicity to the growth and reproduction of *C. elegans*. The in vivo investigations provided substantial evidence supporting the potential of NaYF_4_:Yb Tm NCs as highly effective bioprobes for NIR emission, while exhibiting minimal toxicity [107]. Xing et al. [93] examined the effects of NaYbF_4_ UCNPs in a mouse model. NaYbF_4_ UCNPs were administered to mice at a dose of 150 mg/kg, resulting in the elimination of all UCNPs from the organism after 1 month, as indicated by the absence of fluorescence emanating from NaYbF_4_. Histological examination confirmed that prolonged distribution of UCNPs in vivo did not lead to organ damage or toxic effects [93]. Liu et al. [131] used a one-pot solvothermal process to synthesize PAA-modified BaYbF_5_:Tm nanoparticles. These nanoparticles have been extensively investigated for their potential use as multimodal contrast agents in the gastrointestinal tract. This study highlights its high colloidal stability, moderate cytotoxicity, and negligible hemolysis [131]. Wang et al. [124] examined the impact of UCNPs LaF_3_:Yb, Er on zebrafish to assess the potential toxicity associated with varying concentrations. At concentrations below 100 μg/ml, nanoparticles were found to be nontoxic to zebrafish embryos. However, chronic toxic effects were observed in vivo at 200 μg/ml. These effects include deformities, delayed hatching rates, and impaired embryonic and larval development [124]. Yang et al. [125] investigated the utilization of ^153^Sm-doped Gd (OH)_3_ nanorods as prospective contrast agents for MRI. Studies in Kunming mice have revealed that nanorods primarily accumulate in the liver, spleen, and lungs, with a notably short retention period in these organs. No adverse health effects were observed at a dose of 100 mg/kg throughout the experiment. The accumulation of nanoparticles in organs such as the lungs and heart is minimal and temporary. Histological examination did not reveal any signs of organ damage or lesion [125].

UCNPs generally exhibit lower toxicity, although they can release lanthanide ions that may induce oxidative stress; however, their cytotoxicity is often mitigated through biocompatible surface coatings. In contrast, quantum dots (QDs) are associated with higher toxicity due to the presence of heavy metals like cadmium, which can cause substantial cellular damage. However, it has been revealed that bio-conjugated QDs have a great potencial for improving the effective medication doses to penetrate the BBB and further orient to the target cells inside the brain [132].

CNTs exhibit variable toxicity based on their structure and functionalization, with potential for inflammation and cytotoxicity, particularly upon inhalation. Regarding biodistribution, UCNPs typically accumulate in the liver and spleen, and their modifications can potentially enhance their clearance from the body. QDs also tend to concentrate in the liver and kidneys, raising concerns about long-term retention and chronic toxicity. Different functional groups, such as amines, thiols, and carboxylic acids, can be added to the surface of QDs to facilitate their conjugation with various biomolecules (peptides, antibodies, and nucleic acids). Covalent or noncovalent interactions can be used to conjugate the surface of QDs with targeting ligands, providing tailored therapeutics and selective imaging of the tumor microenvironment or cancer tissues [133]. At the same time, CNTs can penetrate biological barriers and accumulate in various organs, leading to potential long-term health risks. Regulatory challenges differ among these nanoparticles; UCNPs face evolving frameworks due to their novel properties and unknown long-term effects, whereas QDs are heavily regulated because of their toxic components, requiring extensive safety data for biomedical applications. Similarly, CNTs are under increasing scrutiny due to environmental concerns and health risks, necessitating thorough safety evaluations. Table 5 summarizes the key differences in toxicity profiles, biodistribution, and regulatory challenges between UCNPs and other nanoparticles. While UCNPs may present a relatively safer profile for biomedical applications compared to QDs and CNTs, ongoing research into their safety and regulatory considerations is essential for ensuring their responsible use in clinical settings [134–136].

**Table 5.** Table summarizing the key differences in toxicity profiles, biodistribution, and regulatory challenges between UCNPs and other nanoparticles

Nanomaterial	Toxicity profile	Biodistribution	Regulatory challenges	References
Upconversion nanoparticles (UCNPs)	Generally lower toxicity; potential for lanthanide ion release causing oxidative stress. Biocompatible surface coatings often mitigate cytotoxicity.	Accumulate primarily in the liver and spleen; influenced by surface modifications.	Evolving frameworks due to novel properties and limited long-term data.	[35,66,136,183]
Silver nanoparticles (AgNPs)	Higher toxicity; induces oxidative stress and apoptosis; and affects gene expression related to oxidative stress. Accumulates in various organs; hepatobiliary toxicity noted.	Primarily deposited in the mononuclear phagocyte system (MPS); widespread organ distribution was noted.	Heavily regulated due to toxic components; extensive safety data required for biomedical applications.	[184–186]
Gold nanoparticles (AuNPs)	Moderate toxicity; size-dependent effects on cellular response, with smaller sizes causing necrosis or apoptosis. Primarily stored in the liver; lower systemic toxicity compared to AgNPs.	Accumulation in the liver, spleen, kidney, heart, lungs, testis, brain, and thymus; size influences distribution.	Regulatory scrutiny due to potential environmental impact; safety assessments needed.	[187–192]
Copper nanoparticles (CuNPs)	Higher doses are required for toxicity; sex-related differences observed in response. Major accumulation in liver, kidney, and spleen; bio persistence concerns.	Notable accumulation in liver, kidney, and spleen; gastrointestinal exposure leads to higher public risk.	Increasing scrutiny due to health risks associated with copper exposure; requires thorough evaluations.	[193–195]
Titanium dioxide nanoparticles (TiO_2_ NPs)	Size-dependent toxicity; smaller TiO_2_ NPs cause more oxidative stress and DNA damage. Potential lung toxicity upon inhalation; chronic exposure risks identified.	Biodistribution is influenced by size and shape; smaller particles show higher organ distribution	Regulatory concerns regarding inhalation risks and environmental persistence; ongoing assessments are needed.	[196]
Quantum dots (QDs)	Higher toxicity due to heavy metals (e.g., cadmium); can cause significant cytotoxic effects. Toxicity varies based on size, surface chemistry, and concentration.	Tend to accumulate in the liver and kidneys. Long-term retention raises concerns about chronic toxicity.	Heavily regulated due to toxic components (e.g., cadmium). Extensive toxicity data required for approval in biomedical applications.	[197–200]
Carbon nanotubes (CNTs)	Variable toxicity dependent on structure (SWCNTs vs. MWCNTs) and functionalization; can cause inflammation and cytotoxicity. Inhalation can lead to pulmonary toxicity and potential fibrotic responses.	Can penetrate biological barriers and accumulate in various organs, including the lungs. Persistence in biological systems can lead to long-term health risks.	Increasing scrutiny due to environmental impact and potential health risks. Thorough safety evaluations are needed; addressing concerns over environmental impact and potential health risks.	[201–205]

Jang et al. [137] are the first to suggest usage of β-NaYF_4_:Ce, Tb nanophosphors as a potential alternative to QDs. They analyzed the potential toxicity of nanophosphors in embryonic zebrafish and phenotypic characteristics, such as growth retardation, heart deformity, and bent tail. This study highlights that although nano-phosphors are as equally toxic as QDs, the concentration that exerts toxicity in the case of nano-phosphors is 10 times higher than that of QDs. Hence, this nanophosphor can be applied to biomedical imaging over a wide area by optimizing its safe concentration [137]. Sun et al. [138] employed chemistry and molecular biology techniques to synthesize 4 distinct types of upconversion NCs (UCNC-FA, UCNC-Er-FA, UCNC-Tm-FA, and UCNC-Er, Tm-FA) specifically targeted to folate receptors and used as imaging agents. In vivo toxicity tests indicated that these nanoparticles exhibited favorable biocompatibility and minimal toxicity. Histological and hematological examinations of UCNC revealed that UCNC-FA exhibited no observable toxicity in Kunming mice [138]. Vedunova et al. [134] studied the effects of modifying Ln-UCNPs as NaYF_4_:Yb, Tm@NaYF_4_; they observed that nanoparticles cause cellular damage, reduction in calcium ions, and morphological changes in hippocampal cells. In another study, UCNPs were synthesized by coloading platinum (II)-tetraphenyl-tetrabenzoporphyrin and boron dipyrromethene derivative into soybean oil droplets stabilized with bovine serum albumin. Furthermore, the potential toxicity of UCNCs was comprehensively evaluated both in vivo and in vitro. After i.v. injection of 1,200 mg/kg UCNCs, biodistribution analysis revealed that the UCNCs predominantly accumulated in the liver and spleen. Histological, hematological, and blood biochemical tests have shown no signs of toxicity in mice over 60 days after body weight analysis [133]. Chen et al. [139] stated that unmodified Ln-UCNPs can cause nephritis and mild liver toxicity. Unmodified Ln-UCNPs inactivate adenosine triphosphate (ATP) and cause tissue damage by reacting with the phosphate group of ATP [139].

Zhou et al. [5] thoroughly examined the toxicity of silica-coated UCNPs by analyzing the microstructure of Peyer’s patches in the gut and showed that these nanoparticles can cross the intestinal barrier and enter the bloodstream. To further assess the possible toxicity of silica-coated UCNPs, body weight, pathologies, Zn and Cu levels, serum biochemistry, oxidative stress, and inflammatory cytokine levels were examined after 14 days of continuous gavage. The results demonstrated that these nanoparticles did not cause significant toxicity in mice at a dose of 100 mg/kg body weight [5]. Another study by Lay et al. [140] demonstrated the mechanosensitivity and biocompatibility of NaYF_4_:Yb, Er@NaLuF_4_ nanoparticles in *C. elegans*. In vivo and ex vivo experiments were conducted to evaluate potential toxicity. The results showed that these nanoparticles did not show significant toxicity, were optically robust, and did not affect worm production in the digestive tract [140].

Bai et al. [141] have successfully synthesized SiO_2_-coated Ga^3+^-doped ZnO (GZO) nanoparticles with lanthanide ions Yb^3+^ and Tm^3+^ (referred to as Yb/Tm/GZO@SiO_2_). These nanoparticles exhibit the ability to emit red fluorescence, enabling visualization of heart tissue. The toxicity of Yb/Tm/GZO@SiO_2_ UCNPs was examined in vivo, and histological assessments of the relevant organs revealed that these UCNPs exhibited minimal biological toxicity and were suitable as viable fluorescent imaging probes for in vivo applications [141]. Inorganic UCNCs are potential therapeutic and fluorescent diagnostic agents for in vivo applications, such as biological imaging and disease theranostics. These biodegradable UCNCs emit red light based on Yb^3+^/Er^3+^-doped inorganic potassium heptafluozirconate (K_3_ZrF_7_:Yb/Er). Both in vitro and in vivo studies have demonstrated that red-emitting K_3_ZrF_7_:Yb/Er UCNCs have a pH-dependent capacity for biodegradation upon exposure to water. The final biodegradation products can be quickly eliminated from mice with no signs of toxicity [142].

Another study by Asadi et al. [143] investigated the toxic effects of coated and uncoated NaLuF_4_:Yb^3+^, Tm^3+^ UCNPs on blood factors and histopathology in BALB/c mice. The results showed that the uncoated UCNPs were toxic to mice at concentrations of CaCO3@NPs above 250 μg/ml, as evidenced by the decreased red blood cell count and hemoglobin concentration. The coated UCNPs were not toxic to mice, as evidenced by the absence of significant changes in the blood factors and histopathology. The coated UCNPs were also able to accumulate in tumor tissues, suggesting that they could be used for targeted cancer therapy [143]. Samhadaneh et al. [105] demonstrated that Ln-UCNPs possess nontoxic properties and elicit little cellular stress responses. The absence of toxicity was confirmed through in vivo experiments using the model organism *C. elegans* at various concentrations (0 to 100 μg/ml). Inflammatory responses were evaluated using biomarkers, and the results indicated minor cellular stress [105].

Xiang et al. [144] reported a new dual-modal bio-probe PEG-modified Sr_2_YbF_7_: 0.2% Er^3+^/0.8%Tm^3+^ (SYF@PEG) by optimizing the appropriate lanthanides/modifier. SYF@PEG is noteworthy for its ability to effectively provide dual-modal imaging in vitro and in vivo using upconversion luminescence and x-ray computed tomography. It is completely metabolized and excreted by the body. SYF@PEG showed good cell membrane permeability and low toxicity [144]. Hu et al. [145] addressed the challenges of treating Parkinson’s disease (PD) in their research. To successfully target the brain, researchers have modified the surface of UCNPs by altering the red blood cell membrane (RBCM). Stimulating the release of nitric oxide (NO) from NO gas and reducing the amount of proinflammatory molecules in the brain has a light-responsive anti-inflammatory effect. This study showed a reduction in necrosis and apoptosis of dopaminergic neurons in PD mice. Moreover, blood and histological analyses of vital organs (heart, lungs, liver, and kidneys) showed no potential abnormalities. Thus, RBCM/UCMG injections may help reduce PD-related inflammation [145]. Zajdel et al. [146] demonstrated the internalization, biodistribution, and neurotoxicity of NaYF_4_: Yb^3+^ (20%) and Er^3+^ UCNPs in organotypic rat hippocampal slices. Transmission electron microscopy revealed colocalization of UCNPs with specific organelles in neurons and astrocytes. UCNPs enter neurons via caveolae- and clathrin-mediated endocytosis. The toxicity study showed no significant effects on the ultrastructure and cell viability. The localization of UCNPs within specific cell structures and their minimal toxicity profile make them suitable candidates for neurotoxicity investigations and therapeutic interventions [146]. Hosseinifard et al. [147] recently demonstrated the effects of modified NaYF_4_:Yb^3+^, Er^3+^@NaYF_4_ (UCNPs/PAA, UCNPs/PEG-Ner) on plants. The results showed a significant decrease in root number and mass at 100 μg/ml using UCNPs/PAA, whereas UCNPs/PEG-Ner had no effect on seedlings and could neutralize the toxic effects of UCNP. Thus, modifying the surface of UCNPs with PEG-Ner could be a useful tool for plant bioimaging [147]. Yanina et al. [148] studied the effect of SiO_2_-coated UCNPs on photo-induced toxicity. The results showed photo-induced toxicity of SiO_2_-coated UCNPs at 10 mg/ml for 24 h. The phototoxic effect of UCNPs on the human lung-derived fibroblast cell line (FLEH-104) was more pronounced in human laryngeal epidermoid carcinoma (Hep-2) cells, possibly because of differences in the particle internalization efficiency of these cell lines. UCNPs induce apoptosis and autophagy during irradiation of Hep-2 and FLEH-104 cells [148]. Our group reported the first detailed investigation of toxic effects of uncoated core (NaYF_4_:Yb, Er) on gut microbiome and transcriptomic response [119]. In this study, we systematically evaluated the toxicity of uncoated lanthanide-doped UCNP (Ln-UCNP) cores (NaYF_4_, Er) on zebrafish embryos during early development. Ln-UCNPs have been shown to induce several toxic effects, including reduced survival rates, delayed hatching, decreased body length, altered heart rate, impaired blood circulation, and neurobehavioral impairments in response to photoperiod stimulation. Bioimaging studies at 72 hpf revealed that Ln-UCNPs were primarily localized on the chorion, eyes, and skin, whereas after oral ingestion, they accumulated in the pharynx, esophagus, and intestines. Additionally, Ln-UCNP exposure caused disruptions in gut microbiota diversity and abundance, leading to an increase in harmful bacteria in zebrafish. Transcriptomic and Ingenuity Pathway Analysis (IPA) suggested that key signaling pathways such as IL-8, neuroinflammation, cardiac hypertrophy, immune response, and inflammation-related IFN-γ and miR-155 were involved in regulatory effects. A causal network model was constructed, highlighting the significant connections between differentially expressed genes (DEGs), including nitric oxide synthase 2 (nos2) and tumor necrosis factor (tnf), which are associated with adverse outcomes, such as apoptosis, liver damage, and inflammatory responses. RT-qPCR results supported the up-regulation of nos2 and tnf in exposed larvae, consistent with an observed increase in fluorescence-labeled neutrophils and macrophages in lyz: DsRed transgenic zebrafish at 120 hpf, further demonstrating the proinflammatory effects of Ln-UCNPs. In summary, this study elucidated the developmental toxicity, gut microbiota disruption, and proinflammatory impact of Ln-UCNPs on zebrafish, and the causal network generated through IPA provides insight into the potential adverse outcome pathway of Ln-UCNP exposure [119].

In nanotoxicology, computational approaches are increasingly utilized to assess and predict the toxicity of engineered nanomaterials (ENMs), bridging gaps in experimental data and providing insights into the interactions between nanomaterials and biological systems. Computational nanotoxicology encompasses a range of in silico techniques designed to predict the toxicity of nanomaterials based on their physicochemical properties, biological interactions, and environmental behavior. Among these approaches, Quantitative Structure–Activity Relationship (QSAR) models are particularly noteworthy as they predict the toxicity of nanomaterials based on their chemical structure. By analyzing relationships between molecular descriptors and biological activity, QSAR models facilitate risk assessment and help identify potentially harmful nanostructures [149]. These models utilize statistical methods to correlate chemical structure with biological activity, allowing researchers to predict the toxicity of new nanomaterials based on existing data. For instance, recent studies have successfully employed QSAR models to assess the toxicity of various nanoparticles, including metal oxides and carbon nanotubes, demonstrating their potential utility in predicting UCNP toxicity as well.

Another prominent method is molecular dynamics (MD) simulations, which model the behavior of nanomaterials at the atomic level, allowing researchers to examine their interactions with biomolecules such as proteins and lipids. This technique elucidates how the physicochemical properties of nanomaterials influence their biological effects, offering valuable information for designing safer nanomaterials for human applications. MD simulations can provide insights into how nanoparticles interact with cell membranes, proteins, and other biomolecules, which is crucial for understanding their toxicological profiles. These simulations can also help identify critical physicochemical parameters that influence nanoparticle behavior and toxicity, such as size, shape, and surface charge [150]

Additionally, nanoinformatics integrates data from various sources, including experimental results and computational predictions, to characterize the properties and behaviors of nanomaterials, often employing machine learning to analyze large datasets for toxicity predictions [151,152]. Another promising area within computational nanotoxicology is the development of organ-on-a-chip (OOC) systems integrated with computational modeling. These systems allow for real-time monitoring of cellular responses to nanoparticles in a controlled microenvironment that mimics human organs. By combining experimental data from OOC studies with computational models, researchers can enhance the predictive accuracy of toxicity assessments and better understand the underlying mechanisms of nanoparticle-induced effects [153–155].

In silico risk assessment models simulate exposure scenarios to evaluate potential risks associated with nanomaterials, incorporating various biological endpoints and environmental factors to provide a comprehensive risk profile [156]. Furthermore, computational meta-analysis aggregates data from multiple studies to identify trends and draw conclusions about the toxicity of different nanomaterials, enhancing the reliability of predictions by leveraging a broader dataset [157].

Moreover, emerging techniques, such as machine learning, are being applied to analyze large datasets generated from both experimental and computational studies. Machine learning algorithms can identify patterns and relationships within complex datasets that may not be apparent through traditional analysis methods. This capability is particularly valuable in nanotoxicology, where numerous variables can influence outcomes [158].

While specific computational studies on UCNP toxicity are currently lacking, the broader field of computational nanotoxicology offers a wealth of methodologies that can be adapted for future research. By leveraging QSAR models, MD simulations, OOC technologies, and machine learning techniques, researchers can gain deeper insights into the toxicological profiles of UCNPs and other nanomaterials. This comprehensive approach will not only enhance our understanding of nanoparticle interactions with biological systems but also aid in regulatory compliance and designing safer nanomaterials for biomedical applications.

## Biosafety Concern

**Fig. 10.**
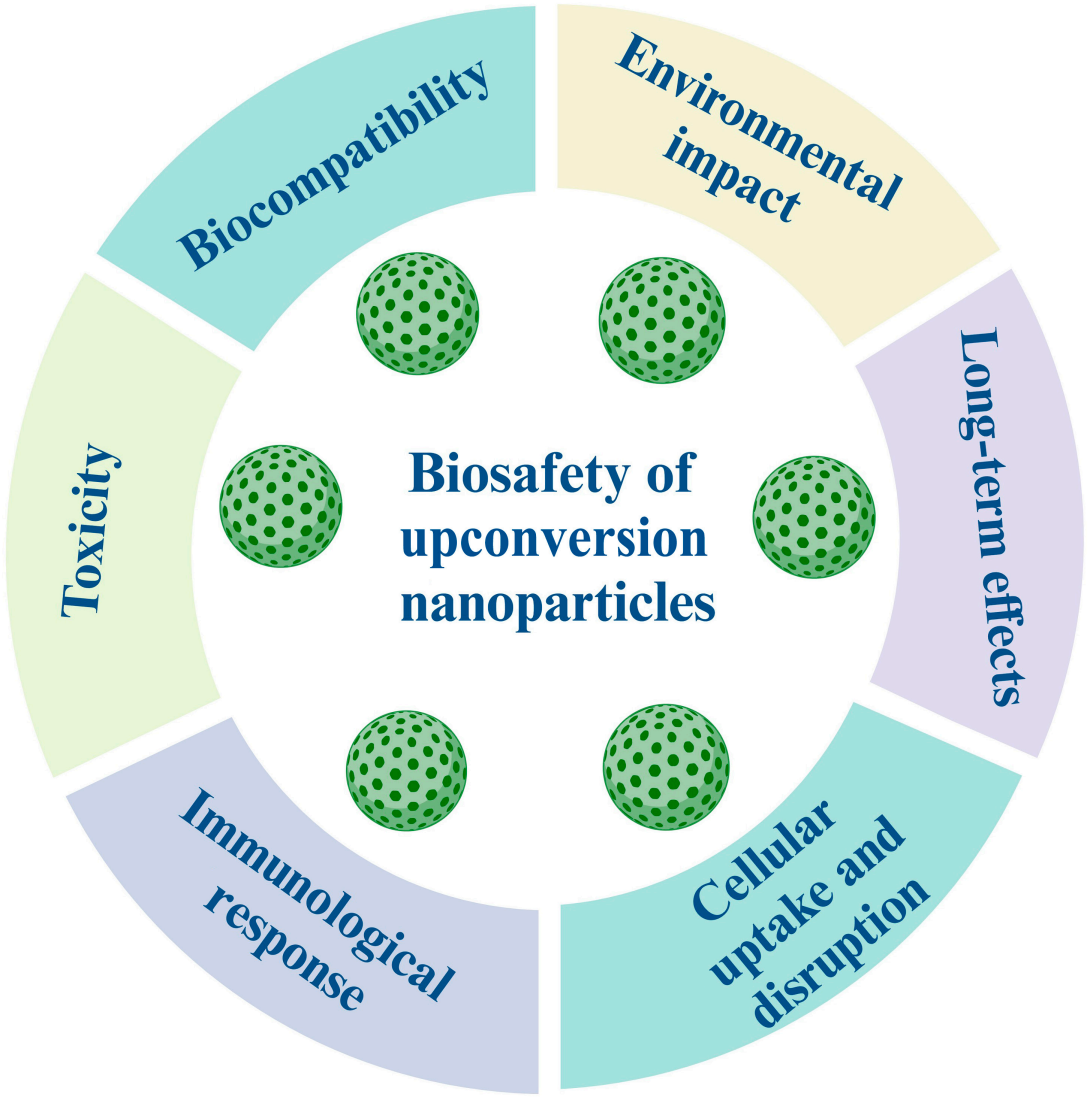
Factors responsible for the biosafety of upconversion nanoparticles.

Nanotechnology and nanomaterials have contributed significantly to economic growth and technical advancement, making them one of the most active and rapidly expanding fields of scientific study and technological development in industrialized nations. However, the distinctive physical and chemical properties of nanoparticles are distinct from those of macromaterials, and they cannot be recognized by traditional procedures and methods, which may cause pollution to human bodies and the ecological environment, compromising human health. Scientists have gradually begun to understand and respect nanomaterials’ potential risks to human health. They also conducted related research in response to concerns regarding potential biological safety issues (Fig. 10). Nanomaterials influence animals at cellular, subcellular, and even protein levels, and studies on their effects and safety have revealed that they are not entirely benign. They can modify fundamental biological functions at the cellular level, such as cell division, proliferation, and apoptosis, as well as control-associated signaling networks. One of the main issues with any nanomaterial is its biocompatibility, specifically how well it interacts with living organisms without harming them. Numerous studies have evaluated the biocompatibility of UCNPs, particularly in vitro and in vivo*,* using various cell lines and animal models.

Nanomaterials have been shown to cause cell death and exhibit cytotoxicity [159,160]. The primary cause of nanomaterial toxicity is the alteration of the physical and chemical properties that occur during the process of material reduction to the nanoscale. For instance, nanomaterials with increased stability are more challenging to break down if applied to or ingested by the human body, and their long-term accumulation in the body negatively affects health. According to research on the biological effects of nanomaterials and their toxicity, nanomaterials can enter the body through the skin, digestive system, and respiratory system to avoid immune system clearance. Once inside the respiratory system, they can precipitate and cause inflammatory reactions that can reduce lung clearance, leading to chronic lung inflammation and oxidative damage. Recent studies have demonstrated a strong correlation between the ability of nanomaterials to induce cell salivation and their biosafety. Additionally, nanomaterials can cross the blood–gas barrier, diffuse from the sedimentation site to nearby tissues, and enter the circulatory system. They can also affect the brain by breaking through the BBB and arousal nerve pathways [161].

Another recent research suggests that UCNPs might have multiple detrimental consequences, such as oxidative stress, inflammation, and cellular damage, potentially resulting in respiratory, cardiovascular, and neurological disorders with extended exposure. The mechanisms underlying UCNP toxicity are intricate and are affected by particle size, surface chemistry, and dosage, indicating that inadequately constructed UCNPs may release harmful ions that lead to cytotoxicity and inflammation [136,162]. A comprehensive strategy is necessary to investigate the long-term effects of UCNP exposure thoroughly. This may entail employing sophisticated in vivo models that accurately replicate human biological responses, such as humanized mouse models or OOC systems that emulate human organ interactions. Furthermore, extensive epidemiological studies evaluating populations exposed to UCNPs via occupational or environmental pathways would yield significant evidence on chronic health effects. In vitro research employing primary human cells can further clarify the mechanisms implicated in UCNP-induced toxicity and inflammation.

Furthermore, integrating high-throughput screening techniques with comprehensive mechanistic investigations will improve our comprehension of UCNP dynamics in biological systems over prolonged durations. Utilizing these methodologies, researchers can more effectively evaluate the long-term health consequences of UCNP exposure and formulate suitable biosafety protocols to reduce any dangers linked to its application in biomedical fields.

The development of nanomedicine is significantly impacted by the toxicity of UCNPs in a number of ways. The appropriate use of UCNPs and other nanomaterial in biomedical and clinical contexts requires careful management of several important issues, such as bioaccumulation, immunological activation, genotoxicity, and clearance. However, the difficulties brought on by UCNP toxicity also offer chances to improve nanomaterials design by applying knowledge gathered from toxicity research to create safer, more intelligent nanomedicines. Through enhancing targeting tactics, biocompatibility, and biodegradability, scientists can lower possible dangers and boost the effectiveness of UCNP-based technologies in imaging, therapy, and diagnostics [163]. For physicochemical and biological characterization, as well as for preclinical and clinical evaluation of UCNPs, the European Upconversion Network has worked to address the need for fixed and validated methodologies (specific or standardized), nano-specific metrology, and UCNP reference materials [24].

The UCNP toxicological investigations offer insightful information that can be used in the broader field of nanomaterials. Researchers can create safer, more biocompatible, and ecologically friendly nanomaterials by understanding the exact mechanisms by which UCNPs produce toxicity, such as size-dependent effects, ROS generation, immunological activation, and biodistribution. This information will be used to develop more environmentally friendly nanotechnologies that optimize the advantages of nanomaterials while lowering any possible hazards to the environment and public health. The primary cause of nanomaterial toxicity is the alteration of the physical and chemical properties that occurs during the process of material reduction to the nanoscale [28]. The toxicity of UCNPs varies according to their size, shape, surface chemistry, dose, and other characteristics. Surface coatings can significantly reduce potential toxicity by enhancing stability and limiting interactions with biological components. However, it is crucial to consider their long-term effects and potential accumulation in tissues. Most studies indicate that appropriately coated UCNPs exhibit low toxicity. Coating with phosphonate provides better biocompatibility for both medical and biological applications. Coating the surface with EDTMP provides the most stable UCNPs that do not produce proinflammatory responses in both in vitro and in vivo studies, thus maintaining the bioimaging intensity [51]. PEI coating is efficient for better drug delivery because it provides optical contrast without background, whereas amphiphilic coating protects UCNPs from dissolution in aqueous media. Citrate coating reduces cytotoxicity and, thus, contributes to the biosafety of UCNPs. PAA-coated UCNPs showed excellent cellular uptake and biodistribution in mice, with no recorded toxicity, proving the use of these UCNPs for long-term therapy and bioimaging in vivo. Another commonly used coating material is silica (SiO_2_), which has favorable properties such as thermodynamic stability, low toxicity, biocompatibility, ease of synthesis, colloidal stability, and scalability. Immune reactions to nanoparticles can affect their effectiveness and safety. Although some studies have noted immunological reactions to UCNPs, it is essential to note that these reactions frequently depend on nanoparticle characteristics, dosage, and administration route.

Employing UCNPs in drug delivery applications can help us analyze the risk–benefit ratio of UCNPs. In situ drug delivery and simultaneous NIR-triggered DOX delivery were achieved by the NaYF4: Yb/Tm@NaGdF4 core-hollow mesoporous silica layer UCNPs in real time monitored by T1-weighted MRI and upconversion luminescence (UCL). UCNP nanocomposites are helpful in a number of studies, including gene targeting in different cancer treatments, controlled drug release, PDT, and photothermal therapy. The primary justification for their application as nanodrugs is the properties of UCNPs that can be exploited in the NIR region [164]. Due to a number of benefits, including deep tissue penetration, low background autofluorescence in biological material, resilience to photobleaching, and minimum photodamage, UCNPs are a superior substitute for conventional optical imaging [165]. Because of these properties, UCNPs have the potential to be used in biology and medicine, particularly in in vitro and in vivo bioimaging [166]. The implications of UCNP-induced toxicity on PDT and biosensing applications are significant. UCNPs can enhance the efficacy of PDT by improving light absorption and energy transfer, but their toxicity may undermine therapeutic outcomes. Toxicity can lead to cellular damage, affecting the viability of target cells and potentially causing adverse effects in surrounding tissues, which could diminish the effectiveness of PDT. In biosensing applications, releasing toxic ions from UCNPs can interfere with biological interactions and signal detection, leading to false results or reduced sensitivity. Moreover, the accumulation of UCNPs in organs raises concerns about long-term biocompatibility and safety, necessitating careful consideration during the design and application of UCNPs in both therapeutic and diagnostic contexts. Improving UCNPs’ quantum yield is essential for uses like sensors and medical care. However, assessing their potential adverse environmental effects is critical, in addition to considering how UCNPs affect human health. The behavior of these nanoparticles and their potential consequences on ecosystems should be evaluated before they are released into the environment. Due to limitations in techniques based on mitochondrial metabolic mobility, such as the inability to distinguish between diving and resting cells, the hazardous potentiality of UCNPs is complex [28].

The outcomes of several safety studies vary depending on the type of UCNP coating, the cell model, and the exposure method, and it is challenging to compare them [167]. Additionally, most studies examined only a small range of concentrations and exposure durations and thus failed to consider the products of UCNP degradation and transformation in the biochemical environment [168]. The UCNP toxicological investigations offer insightful information that can be used in the larger field of nanomaterials. Researchers can create safer, more biocompatible, and ecologically friendly nanomaterial by understanding the exact mechanisms by which UCNPs produce toxicity, such as size-dependent effects, ROS generation, immunological activation, and biodistribution. This information will be used to develop more environmentally friendly nanotechnologies that optimize the advantages of nanomaterial while lowering any possible hazards to the environment and public health.

Studies reveal that UCNPs can activate DCs, which, upon cellular interaction, release proinflammatory cytokines such as IL-6, TNF-α, and IL-1β. In addition to playing essential roles in the process of mediating inflammation, these cytokines have the ability to set off a chain reaction of immunological responses that, if not adequately managed, could lead to damaging of the tissue [127,128,130]. For example, IL-6 is particularly important since it not only contributes to the development of inflammation but also has an effect on the differentiation of T cells, which can potentially exacerbate tissue injury [169]. Furthermore, research has demonstrated that the surface chemistry of UCNPs bears the potential to influence both their toxicity and their inflammatory response. It is possible that poorly engineered UCNPs could result in ion leakage and increased oxidative stress, which would, therefore, activate inflammatory pathways [170]. A solid understanding of these mechanisms is necessary to build efficient biosafety methods. Researchers will be able to advise the design of safer nanoparticles better and optimize their therapeutic applications while simultaneously minimizing unfavorable immune responses if they can disentangle the individual inflammatory pathways that are involved in the toxicity that UCNP generates. In addition to facilitating the establishment of guidelines for the safe application of UCNPs in clinical settings, this all-encompassing approach will help us better understand how UCNPs interact with biological systems.

When designing a new biological application of UCNPs, the EQS for end users are crucial factors from a regulatory and industrial perspective. To comply with the most stringent regulatory requirements for the commercialization of UCNPs, it is advised to contact the national regulatory bodies as soon as the intellectual property rights for the biomedical application have been obtained [171]. Regulatory initiatives for ENMs, including UCNPs, vary significantly across the European Union (EU), the United States, and Australia. In the EU, the regulatory framework is primarily governed by the REACH (Registration, Evaluation, Authorisation and Restriction of Chemicals) regulation, which requires extensive safety data on nanomaterials. The European Medicines Agency also provides guidelines for medicinal products containing nanomaterials, emphasizing risk assessment and post-market surveillance. However, the EU’s approach has been criticized for being overly complex and slow to adapt to rapid technological advancements in nanotechnology. In contrast, the US regulatory landscape is managed by the Food and Drug Administration (FDA) and the Environmental Protection Agency. The FDA has issued guidance on the evaluation of nanotechnology products, but it lacks a comprehensive regulatory framework specifically tailored for nanomaterials. This has led to concerns about inconsistent oversight and potential gaps in safety assessments [35,172]. The US approach tends to focus more on pre-market evaluations rather than ongoing post-market monitoring, which can leave significant risks unaddressed.

Australia’s Therapeutic Goods Administration (TGA) has also established guidelines for nanomaterials, aligning closely with EU regulations while incorporating elements from the US system. The TGA emphasizes a risk-based approach but has been criticized for its reactive stance rather than proactive measures in regulating emerging technologies like UCNPs [173–175].

Despite these efforts, critical gaps remain in current guidelines across all 3 regions. There is a lack of standardized definitions and classifications for nanomaterials, which complicates regulatory compliance and enforcement issues. Additionally, existing frameworks often do not adequately address the unique properties and behaviors of UCNPs, particularly concerning their long-term environmental impact and potential health risks. This inconsistency underscores the urgency of establishing standardized global regulations to ensure comprehensive safety assessments and harmonized oversight of nanotechnology products [35,176].

The regulatory authorities of the European Union, United States, and Australia deem existing regulations adequate for the effective toxicological evaluation of nano products, although numerous initiatives are being pursued at both national and international levels to identify and assess risks and to implement new regulations if necessary. However, in the past few years, the regulatory bodies of several countries have considered integrating new regulatory guidelines for nanomaterials and nanotechnology products at national levels. For instance, the United States created the National Nanotechnology Initiative to expand nanotechnology applications; Australia established the National Nanotechnology Strategy to advance the nanotechnology field, simultaneously addressing the issues affecting efficacious and accountable expansion of nanotechnology. Technical jargons should be simplified and balanced information on the risks and benefits of UCNPs should be presented to policymakers and the public to effectively communicate the potential dangers to policymakers and the public. While the regulatory initiatives in the EU, United States, and Australia aim to address the safety of ENMs, significant gaps persist that hinder effective regulation. A coordinated global approach is essential to mitigate risks associated with UCNPs and other nanomaterials in order to protect public health and the environment effectively.

## Strategies to Minimize UCNP Toxicity

While research on UCNPs, including cellular uptake, cytotoxicity, stability, surface load, biodistribution, and dose effects within living animals, holds significant implications for their future clinical applications, the transition from laboratory to routine clinical use is not without challenges [177]. The most important ones are biocompatibility and toxicity assessments, which require a thorough understanding of how UCNPs interact with biological systems over extended periods of time to ensure patient safety.

Numerous methods have been used to reduce UCNP toxicity. These include surface modification with biocompatible coatings, regulation of UCNP size and shape, utilization of biodegradable materials, selection of inert and safe core materials, thorough toxicity assessments, and monitoring of dosage and exposure [89]. Combining these strategies can improve the biocompatibility and safety profile of UCNPs, thereby enabling their secure implementation in biomedical applications. A recent study by Alkahtani et al. [178] suggested that engineered UCNPs in the core–shell–shell (CSS) structure, with optimized dopant ion concentrations, exhibited low cytotoxicity in mice at the tested concentration. This study examined the cytotoxicity of UCNPs in mice using engineered bright and small UCNP in a CSS structure, improving the biological transparency window under biocompatible excitation wavelengths by optimizing dopant ion concentrations. They discovered that UCNPs were not harmful to mice at a concentration of 100 mg/kg body weight. Additionally, the UCNPs were evenly dispersed throughout the body and were well-tolerated. Furthermore, it was observed that the UCNPs were eliminated from the urine and feces [178]. Another study showed that covering UCNPs with a phosphonate layer can increase their chemical stability under physiological conditions. This is because phosphonates, which are molecules that may bind to metal ions, can aid in preventing the release of harmful ions from UCNPs. This may reduce the cytotoxicity of the UCNPs [179]. Thus, the UCNPs’ surface functional group also plays a crucial role in making the UCNPs surface hydrophilic or modifiable, thereby reducing toxicity [180].

However, nanoparticles might not be coated as the coating method’s efficacy has not yet been established. Such coatings’ performance in challenging physiological settings is erratic and contingent on a number of variables. UCNP cytotoxicity is often affected by surface modification [5]. It is vital to conduct a critical analysis of the limitations of animal models utilized in the toxicity studies of UCNPs to gain knowledge of the relevance of these models to human reactions. Prior research has documented the biodistribution patterns, absorption and excretion processes, and short-, subchronic, and long-term toxicities of some UCNPs in live species, including mice (*Mus musculus*), zebrafish (*Danio rerio*), and nematodes (*C. elegans*) [77,99,107,119,133,136].

Each of these aforementioned species has its own set of benefits and drawbacks. Zebrafish are frequently used because their embryos are transparent, enabling researchers to observe nanoparticle interactions and toxicity in real time. However, their immune systems are somewhat different from those of humans, which may make it difficult to extrapolate the inflammatory responses seen in these models to the physiology of humans. Rodents, including mice and rats, have genetic and physiological parallels to humans and are utilized in various applications. The information that they provide regarding the systemic toxicity and biodistribution of UCNPs is extremely significant. Nevertheless, rodent models can still fall short in accurately reproducing human immune responses and may not fully reflect the long-term effects of persistent exposure to nanoparticles. *C. elegans* is a simple model organism that enables high-throughput screening of toxicity. However, when it comes to examining inflammatory pathways that are relevant to UCNP-induced toxicity, its relevance is limited because it does not possess a complicated immune system [35,107,119,136].

Researchers should consider the possibility of inventing or adopting new models that more accurately reflect human biology to improve the relevance of toxicity studies. Emerging alternative models such as OOC systems and humanized mice models are gaining traction in biomedical research, particularly for studying complex biological processes and therapeutic responses. These innovative approaches provide significant advantages in mimicking human physiological responses and addressing the complexities of nanoparticle interactions, ultimately enhancing the reliability and relevance of preclinical studies. They aim to address the limitations of traditional in vivo models, especially in the context of UCNP exploration.

OOC technology involves microfluidic devices that contain living cells cultured under conditions that mimic the physiological environment of human organs. These systems can replicate organ-level functions and interactions, providing a more relevant platform for studying human biology compared to conventional animal models [155,181]. OOC systems utilize human cells, which enhance their predictive accuracy regarding human physiological responses. This is particularly important as animal models often fail to accurately predict human outcomes due to species differences. The use of OOC models reduces reliance on animal testing, addressing ethical concerns surrounding animal research. This aligns with societal shifts toward more humane scientific practices. By fluidically coupling multiple organ chips, researchers can create body-on-chip systems that mimic whole-body physiology, allowing for the study of inter-organ interactions and drug metabolism in a controlled environment [155,181].

Despite their advantages, OOC systems face challenges, such as high initial setup costs and the need for standardization in protocols to gain acceptance from regulatory bodies and the pharmaceutical industry [155,181]. Additionally, while they excel in modeling specific organ functions, they may not fully replicate the complexity of whole organisms.

Humanized mice models are another interesting approach to mitigate the limitations of current in vivo animal models. These mice are genetically engineered to express human genes or tissues, allowing researchers to study human-specific biological processes and diseases within a living organism. These models are particularly useful for understanding immune responses and drug interactions in a context that more closely resembles human physiology than traditional mouse models. Humanized mice provide an in vivo platform to study the effects of therapies and disease mechanisms that cannot be effectively modeled in vitro. These models can be tailored to represent various human diseases, facilitating the study of disease progression and treatment responses in a controlled setting [155,181]. The primary limitations of humanized mice include variability in how well they mimic human biology, potential ethical concerns regarding genetic modifications, and the complexity of maintaining these models. Moreover, they still rely on traditional animal research frameworks, which may not always translate findings effectively to humans [155,181].

The combination of OOC technology with humanized mice could lead to integrative research strategies that leverage the strengths of both platforms. For instance, initial screenings could be conducted using OOC systems before validating findings in humanized mice, creating a more robust pipeline for drug development. OOC systems and humanized mice models represent significant advancements in biomedical research methodologies. By addressing the limitations inherent in traditional animal models, these alternatives enhance our ability to explore complex biological interactions and improve therapeutic strategies involving UCNPs. As these technologies continue to evolve, they hold promise for more effective and ethical approaches to medical research [155,181,182].

Researchers have the ability to increase the prediction value of UCNP toxicity studies and contribute more effectively to the development of biosafety policies if they address these constraints and investigate novel modeling methodologies.

## Future Perspectives and Conclusion

UCNPs offer promising applications across various biomedical fields, such as imaging, targeted drug delivery, and PDT. Despite their remarkable potential, the successful translation of UCNPs into clinical practice is hindered by critical challenges, including toxicity, biocompatibility, and long-term safety concerns. Addressing these issues through targeted research and innovation is essential to unlock their full potential. Future research must prioritize optimizing surface coatings to enhance biocompatibility and reduce toxicity. Quantitative comparisons of materials such as PEG, silica, and hybrid coatings are needed to identify the most effective strategies. Additionally, improving targeting efficiency through active (e.g., ligand-specific functionalization) and passive mechanisms (e.g., enhanced permeability and retention effect) is crucial to maximize therapeutic efficacy while minimizing off-target effects.

The development of advanced toxicity assessment methodologies, including high-throughput screening and in silico simulations, will accelerate hazard identification and provide mechanistic insights into UCNP-induced toxicity. These approaches will enable early risk detection and guide the design of safer nanomaterials. Furthermore, long-term biodistribution studies are essential to evaluate the bio-persistence, clearance, and cumulative effects of UCNPs on human health and the environment. A deeper understanding of the molecular pathways underlying UCNP toxicity will further inform risk assessment and mitigation strategies. Rigorous clinical validation through well-designed trials is imperative to establish the safety and efficacy of UCNPs in diverse biomedical applications. Standardizing toxicity evaluation protocols and harmonizing regulatory frameworks specific to UCNPs will ensure consistent and reliable safety assessments. Ethical considerations, such as addressing privacy concerns, obtaining informed consent, and transparently communicating potential risks, are equally critical to fostering public trust and ensuring the responsible development of UCNP-based technologies. A collaborative and interdisciplinary approach combine expertise from toxicology, materials science, and biomedical research, which is essential to bridge existing knowledge gaps. By fostering synergistic interactions among these fields, researchers can leverage advancements in nanomaterial design, biological safety assessment, and therapeutic innovation to overcome current limitations. This approach will enable the development of robust frameworks for evaluating UCNP safety, optimizing their performance, and accelerating their transition from bench to bedside.

In summary, while UCNPs hold immense promise for revolutionizing biomedical applications, addressing challenges related to biocompatibility, scalability, targeting efficiency, clinical validation, and ethical considerations is paramount. By systematically tackling these issues, researchers can unlock the full potential of UCNPs, ensuring their safe and effective integration into clinical practice while minimizing risks to human health and the environment. The future of UCNPs lies in a balanced approach that combines innovation, rigorous safety evaluation, and ethical responsibility.

## Key Limitations

This review on UCNP toxicity and biodistribution offers valuable insights through bibliometric analysis (2008 to 2024) and presents several key limitations. First, its primary reliance on the Web of Science may introduce geographic and linguistic biases by overlooking studies in other databases or languages. Second, the analysis of the molecular-level interactions between the physicochemical properties of UCNPs and biological systems could be more comprehensive. Third, the lack of standardized toxicity protocols complicates the cross-study comparisons. Fourth, while in vitro studies are emphasized, a greater focus on long-term in vivo effects would enhance clinical relevance. Fifth, the predominantly biomedical focus leaves environmental impacts unexplored. Finally, exclusion criteria based on language or document type may have affected the review’s inclusiveness. Addressing these limitations through expanded database searches, standardized assessment methods, and the incorporation of more in vivo and ecotoxicological data in future studies would significantly advance our understanding of UCNP safety profiles and facilitate their responsible development for biomedical and environmental applications.
